# Iron-Binding and Anti-Fenton Properties of Novel Amino Acid-Derived Cyclic Imide Dioximes

**DOI:** 10.3390/antiox8100473

**Published:** 2019-10-11

**Authors:** Janez Mravljak, Žiga Jakopin

**Affiliations:** Faculty of Pharmacy, University of Ljubljana, Aškerčeva 7, SI-1000 Ljubljana, Slovenia; janez.mravljak@ffa.uni-lj.si

**Keywords:** amino acid, building block, iron(II)-chelating agents, cyclic imide dioxime, antioxidant, Fenton reaction, anti-Fenton

## Abstract

We present a novel route for the preparation of amino acid-derived cyclic imide dioxime derivatives. Readily accessible amino acids were conveniently converted to their corresponding cyclic imide dioximes in simple synthetic steps. The aim of this work was to describe and compare the iron-chelating and antioxidant properties of synthesized compounds in relation to their molecular structure, and in particular, which of those features are essential for iron(II)-chelating ability. The glutarimide dioxime moiety has been established as an iron(II)-binding motif and imparts potent anti-Fenton properties to the compounds. Compound **3** was shown to strongly suppress hydroxyl radical formation by preventing iron cycling via Fe-complexation. These findings provide insights into the structural requirements for achieving anti-Fenton activity and highlight the potential use of glutarimide dioximes as antioxidants.

## 1. Introduction

**Figure 1:**
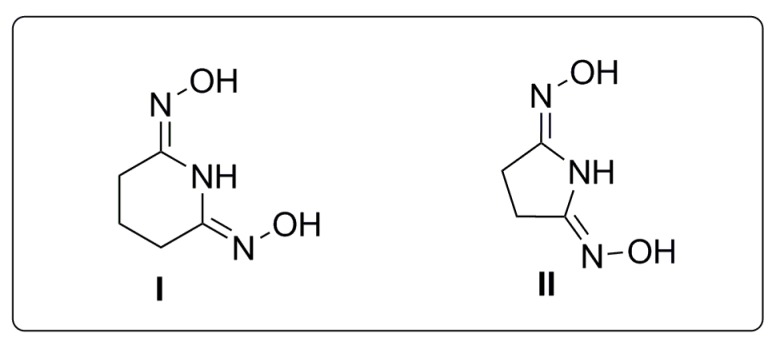
Basic cyclic imide dioxime scaffolds.

Cyclic imide dioximes, such as glutarimide dioxime (**I**; also known as 2,6-dihydroxyiminopiperidine) [1] and succinimide dioxime (**II**; also known as 2,5-dihydroxyiminopyrrolidine) [2] (shown in Figure 1), have long been known for their ability to form complexes with metals [3,4,5,6,7]. Cyclic imide dioximes are commonly typified by a tridentate binding site [8] and have already been evaluated for their major transition metal chelating abilities [9,10]. **I**, for example, is able to bind Fe(III) and subsequently form a strong complex [3a]. The solved crystal structures of the complex revealed 1:1 and 1:2 metal-ligand complex with tridentate coordination in which the ligand coordinates to iron through the two oxime oxygen atoms and the imide nitrogen atom. The bond lengths of Fe-O and Fe-N as well as the high ionic charge and small radius of Fe(III) are of particular importance in determining the order of binding strength when compared to other tested metal ions. The binding strength of **I** with these ions follows the order: Fe(III) > Cu(II) > Pb(II) > Ni(II) [3]. It was suggested that if grafted onto a solid substrate, its complexing ability could be even greater [11]. Several imide dioxime derivatives, such as phthalimide dioxime and naphthalimide dioxime, have recently been described [10,12]. Interestingly, the imide dioxime functionality has also found use in the synthesis of pharmaceutically active compounds [13].

**Figure 2:**
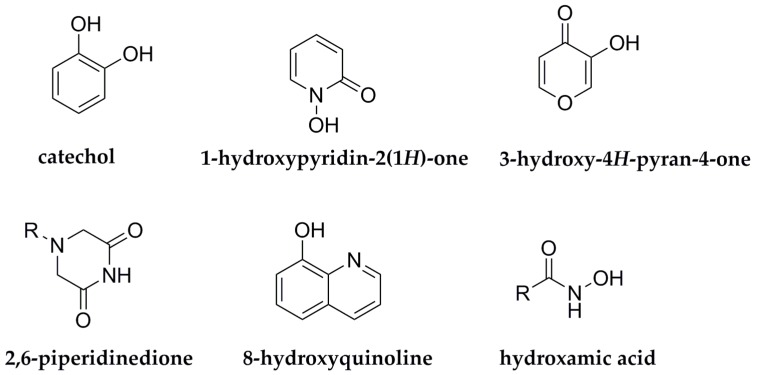
Several examples of different iron-binding motifs [14,16].

In biological systems, reactive oxygen species (ROS) often originate from the interaction of redox-active metal ions with superoxide and/or hydrogen peroxide (Fenton reaction) consequently leading to oxidative damage and contribute to neurodegenerative disorders like Alzheimer’s and Parkinson’s. Free iron (either as Fe(II) or Fe(III)) is therefore toxic even at very low concentrations [14]. Its chelation is also beneficial in cases of thalassemia to neutralise the iron overload in the body [15]. Given the fact that Fenton chemistry depends on the oxidation of redox-active metal ions in their reduced form (e.g., Fe(II) or Cu(I)) with hydrogen peroxide to generate hydroxyl radicals, the redox chemistry of these elements is closely regulated. Thus, contrary to the common antioxidants, which inactivate ROS only after they have been formed, by the chelation of these metal ions with chelating agents, the formation of ROS could be prevented. For example, quercetin belongs to the flavonoid class of polyphenolic antioxidants whose antioxidant properties have been shown to rely mostly on preventing the Fenton chemistry via chelation, and the subsequent sequestration, of iron(II) [16]. Extensive research has been carried out in recent years on the design of iron chelators and pharmacophore conjugates affording molecules with multimodal functions (selected iron-binding motifs are depicted in Figure 2) [17].

These findings prompted us to design novel imide dioximes with potential iron(II)-chelating abilities. In the search for a synthetic route to novel cyclic imide dioximes, we used readily accessible amino acids which were converted to the corresponding cyclic imide dioximes in simple synthetic steps. The designed and synthesized cyclic imide dioximes are amenable to integration into larger structures or onto resins, either directly or via a linker. The present study was conducted with a focus on the complexation of iron(II). The chelating effect of the synthesized compounds on ferrous ions was established by the ferrozine assay and compared to those of quercetin and ethylenediaminetetracetic acid (EDTA). The aim of this work was to describe and compare the iron-chelating and antioxidant properties of synthesized compounds in relation to their molecular structure, in particular, which of those features are essential for iron(II)-chelating ability. Finally, the selected compounds were screened for their anti-Fenton properties by determining their ability to halt copper and iron redox cycling via metal complexation in the ascorbate redox system assay.

## 2. Materials and Methods

### 2.1. Preparation of Cyclic Imide Dioximes

Chemicals were obtained from Acros, Sigma-Aldrich and TCI, and used without further purification. Succinimide dioxime and glutarimide dioxime were synthesized as previously described [3,18]. Analytical thin layer chromatography (TLC) was performed on Merck 60 F254 silica gel plates (0.25 mm), using visualization with ultraviolet light and ninhydrin. Column chromatography was carried out on silica gel 60 (particle size 240–400 mesh). Melting points were determined on a Reichert hot stage microscope and are uncorrected. ^1^H- and ^13^C-NMR spectra were recorded at 400 MHz and 100 MHz, respectively, on a Bruker AVANCE III spectrometer in DMSO-d_6_ or CDCl_3_ solution with tetramethylsilane (TMS) as internal standard. Spectra were assigned using gradient correlation spectroscopy (COSY) and heteronuclear single quantum coherence (HSQC) spectroscopy experiments. IR spectra were recorded on a Perkin-Elmer 1600 FT-IR spectrometer. Mass spectra were obtained using a VG-Analytical Autospec Q mass spectrometer. Microanalyses were performed on a 240 °C Perkin-Elmer CHN analyzer.

#### 2.1.1. Chemistry

**Scheme 1:**
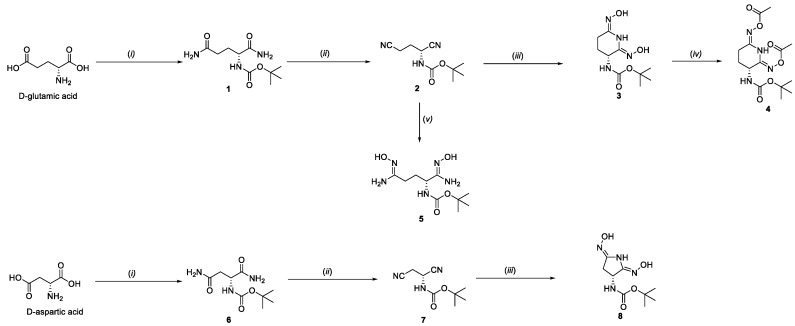
Reagents and conditions: (i) (a) (Boc)_2_O, Na_2_CO_3_, dioxan/water; (b) (Boc)_2_O, (NH_4_)_2_CO_3_, pyridine, tetrahydrofuran, rt; (ii) cyanuric chloride, DMF, first at 0 °C then at rt; (iii) NH_2_OH × HCl, Et_3_N, EtOH, reflux; (iv) AcCl, diisopropylethylamine, CH_2_Cl_2_, first at 0 °C then at rt; (v) NH_2_OH × HCl, Na_2_CO_3_, EtOH, H_2_O, rt.

**Scheme 2:**
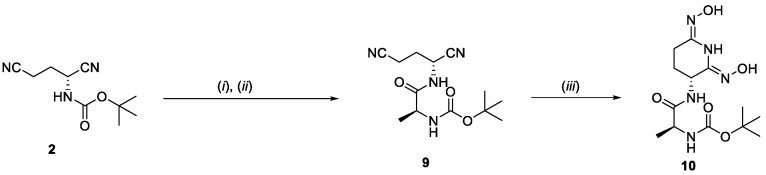
Reagents and conditions: (i) TFA/CH_2_Cl_2_ (1:5); (ii) Boc-L-Ala, TBTU, diisopropylethylamine, CH_2_Cl_2_; (iii) NH_2_OH × HCl, Et_3_N, EtOH, reflux.

The synthesis of novel amino acid-derived cyclic imide dioximes has been carried out as shown in Scheme 1 and Scheme 2.

##### Conversion of Amides to Nitriles

To a stirred solution of amide (1.0 mmol) in dry DMF (5 mL), cyanuric chloride (1 mmol) was added at 0 °C. After stirring for 15 minthe mixture was allowed to warm to room temperature and stirring then continued for 3 h. The reaction mixture was quenched with water (30 mL) and extracted with ethyl acetate (3 × 50 mL). The organic phase was washed with 10% citric acid (2 × 10 mL), water (20 mL), saturated NaHCO_3_ solution (2 × 10 mL), water (20 mL) and then dried over anhydrous Na_2_SO_4_. The solvent was removed under reduced pressure and the residue was additionally purified by flash chromatography on a silica gel column using chloroform/methanol (20:1) as eluant to afford the corresponding nitrile.

##### Synthesis of Cyclic Imide Dioximes

To a stirred solution of nitrile (1.0 mmol) and hydroxylamine hydrochloride (2.5 mmol) in ethanol, triethylamine (1.5 mmol) was added. The mixture was then heated to reflux and stirred overnight. The solvent was removed under reduced pressure and the residue was additionally purified by flash chromatography on a silica gel column if necessary using chloroform/methanol (9:1) as eluant to afford the corresponding amidoxime.

##### General Procedure for Bisamidoxime Synthesis

To a stirred solution of nitrile (1.0 mmol) and hydroxylamine hydrochloride (2.5 mmol) in a mixture of ethanol and water, sodium carbonate (2.5 mmol) was added. The mixture was then stirred overnight at room temperature. The solvent was removed under reduced pressure and the residue was additionally purified by flash chromatography on a silica gel column if necessary using chloroform/methanol (9:1) as eluant to afford the corresponding amidoxime.

#### 2.1.2. Characterization of Compounds

##### *Tert*-butyl (1*R*)-(1,5-diamino-1,5-dioxopentan-2-yl)carbamate (1)

White amorphous powder, yield: 79%; m.p. 128–131 °C. ^1^H-NMR (DMSO-d_6_, 400 MHz): δ = 1.38 (s, 9H, 3CH_3_), 1.62–1.72 (m, 1H, CH_2A_-β-Glu), 1.78–1.87 (m, 1H, CH_2B_-β-Glu), 2.08 (t, 2H, *J* = 6.8 Hz, CH_2_-γ-Glu), 3.79–3.83 (m, 1H, CH-Ala), 6.76–6.78 (m, 2H, CONH_2_), 6.98 (s, 1H, CONH_2A_), 7.24–7.28 (m, 2H, CONH_2B_ and NHBoc) ppm.

##### *Tert*-butyl (1*R*)-1,3-dicyanopropylcarbamate (2)

Synthesized according to the *General procedure for the conversion of amides to nitriles*. White amorphous powder, yield: 87%; m.p. 93–94 °C. 1H-NMR (DMSO-d_6_, 400 MHz): δ = 1.41 (s, 9H, 3CH_3_), 2.00–2.11 (m, 2H, CH_2_-β-Glu), 2.59 (t, 2H, *J* = 7.2 Hz, CH_2_-γ-Glu), 4.49–4.56 (m, 1H, CH), 7.80 (d, 1H, *J* = 6.0 Hz, NH) ppm. MS (ESI): *m/z* (%) = 210.1 (M+H)^+^. IR (KBr): *ν* = 3358, 2984, 2941, 2258, 1685, 1517, 1372, 1340, 1311, 1280, 1158, 1057, 875, 604 cm^−1^. HRMS Calcd for C_10_H_16_N_3_O_2_ *m/z:* 210.1250 (M+H)^+^, found 210.1243. Anal. Calcd for C_10_H_15_N_3_O_2_ (%): C, 57.40; H, 7.23; N, 20.08. Found: C, 57.38; H, 7.40; N, 19.94. [α]_D20_ = +102.6° (*c* 0.12, CH_2_Cl_2_).

##### *Tert*-butyl (3*R*)-2,6-bis(hydroxyimino)piperidinylcarbamate (3)

Synthesized according to the *General procedure for the synthesis of cyclic imide dioximes*. Beige amorphous powder, yield: 83%; m.p. 189–193 °C. ^1^H-NMR (DMSO-d_6_, 400 MHz): δ = 1.39 (s, 9H, 3CH_3_), 1.67–1.78 (m, 1H, CH_2A_-β), 1.82–1.88 (m, 1H, CH_2B_-β), 2.41–2.44 (m, 2H, CH_2_-γ), 4.37–4.38 (m, 1H, CH), 7.19 (d, 1H, *J* = 8.8 Hz, Boc-NH), 8.29 (s, 1H, NH), 9.94 (s, 1H, OH), 10.28 (s, 1H, OH) ppm. ^13^C-NMR (DMSO-d_6_, 100 MHz): δ = 22.5 (CH_2_-β), 26.3 (CH_2_-γ), 28.2 (C(CH_3_)_3_), 46.1 (CH), 77.9 (C(CH_3_)_3_), 143.9 (C=N), 144.8 (C=N), 155.0 (CO) ppm. MS (ESI): *m*/*z* (%) = 259.1 (M+H)^+^. IR (KBr): ν = 3401, 3349, 2981, 2932, 2363, 1662, 1521, 1391, 1312, 1246, 1166, 1050, 1022, 982, 960, 927, 855, 779 cm^−1^. HRMS Calcd for C_10_H_19_N_4_O_4_ *m*/*z*: 259.1406 (M+H)^+^, found 259.1398. Anal. Calcd for C_10_H_18_N_4_O_4_ (%): C, 46.50; H, 7.02; N, 21.69. Found: C, 46.78; H, 6.80; N, 21.28. [α]_D_^20^ = +5.7° (*c* 0.16, CH_2_Cl_2_).

##### *Tert*-butyl ((*R*,2*Z*,6*Z*)-2,6-bis(acetoxyimino)piperidin-3-yl)carbamate (4)

Synthesized according to the *General procedure for the acetylation*. Light yellow oil, yield: 71%; ^1^H-NMR (CDCl_3_, 400 MHz): δ = 1.42 (s, 9H, 3CH_3_), 1.81–1.91 (m, 1H, CH_2_-β), 2.13-2.16 (m, 1H, CH_2_-β), 2.19 (s, 3H, CH_3_CO), 2.21 (s, 3H, CH_3_CO), 2.57–2.66 and 2.77–2.83 (2m, 1H each, CH_2_-γ), 4.49–4.54 (m, 1H, CH), 5.33 (d, 1H, *J* = 6.4 Hz, Boc-NH), 8.34 (s, 1H, NH) ppm. ^13^C-NMR (CDCl_3_, 100 MHz): δ = 19.5 (2×CH_3_), 23.4 (CH_2_-β), 26.0 (CH_2_-γ), 28.3 (C(CH_3_)_3_), 30.9, 48.0 (CH), 80.6 (C(CH_3_)_3_), 148.5 (C=N), 149.0 (C=N), 155.1 (CO), 167.2 (2×CO) ppm. MS (ESI): *m*/*z* (%) = 343.2 (M+H)^+^. IR (KBr): ν = 3313, 2975, 1762, 1646, 1518, 1368, 1205, 1163, 1059, 1006, 921, 852 cm^−1^. HRMS Calcd for C_14_H_23_N_4_O_6_ *m*/*z*: 343.1618 (M+H)^+^, found 343.1631. Anal. Calcd for C_14_H_22_N_4_O_6_ (%): C, 49.12; H, 6.48; N, 16.37. Found: C, 49.40; H, 6.43; N, 16.00. [α]_D_^20^ = +0.2° (*c* 0.18, MeOH).

##### *Tert*-butyl ((*R*,1*Z*,5*Z*)-1,5-diamino-1,5-bis(hydroxyimino)pentan-2-yl)carbamate (5)

Synthesized according to the *General procedure for the synthesis of bisamidoximes*. Beige amorphous powder, yield: 74%; m.p. 126–130 °C. ^1^H-NMR (DMSO-d_6_, 400 MHz): δ = 1.38 (s, 9H, 3CH_3_), 1.69–1.79 (m, 2H, CH_2_-β-Glu), 1.89–1.91 (m, 2H, CH_2_-γ-Glu), 3.89 (q, 1H, *J* = 8.0 Hz, CH), 5.25 (s, 2H, NH_2_), 5.33 (s, 2H, NH_2_), 6.80 (d, 1H, *J* = 9.2 Hz, Boc-NH), 8.73 (s, 1H, OH), 8.96 (s, 1H, OH) ppm. ^13^C-NMR (DMSO-d_6_, 100 MHz): δ = 28.1 (CH_2_-β), 28.7 (C(CH_3_)_3_), 30.8 (CH_2_-γ), 51.6 (CH), 78.5 (C(CH_3_)_3_), 152.8 (C=N), 153.3 (C=N), 155.3 (CO) ppm. MS (ESI): *m*/*z* (%) = 276.2 (M+H)^+^. IR (KBr): ν = 3480, 3365, 2978, 2368, 1675, 1521, 1363, 1248, 1172, 1046, 949, 922, 834, 702 cm^−1^. HRMS Calcd for C_10_H_22_N_5_O_4_ *m*/*z*: 276.1672 (M+H)^+^, found 276.1664. Anal. Calcd for C_10_H_21_N_5_O_4_ (%): C, 43.63; H, 7.69; N, 25.44. Found: C, 43.91; H, 7.82; N, 25.38. [α]_D_^20^ = +2.1° (*c* 0.13, CH_2_Cl_2_).

##### *Tert*-butyl (1*R*)-3-amino-1-(aminocarbonyl)-3-oxopropylcarbamate (6)

White amorphous powder, yield: 85%; m.p. 190–192 °C. ^1^H-NMR (DMSO-d_6_, 400 MHz): δ = 1.38 (s, 9H, 3CH_3_), 2.31–2.43 (m, 2H, CH_2_-β-Asp), 4.13–4.19 (m, 1H, CH), 6.80 (d, 1H, *J* = 8.8 Hz, NH), 6.89 (s, 1H, CONH), 6.99 (s, 1H, CONH), 7.18 (s, 1H, CONH), 7.27 (s, 1H, CONH) ppm. MS (ESI): *m/z* (%) = 232.1 (M+H)^+^. IR (KBr): ν = 3392, 3341, 3196, 2983, 2936, 2364, 1525, 1408, 1368, 1327, 1275, 1248, 1175, 1057, 1029, 950, 858, 814, 770 cm^−1^. HRMS Calcd for C_9_H_18_N_3_O_4_ *m*/*z*: 232.1297 (M+H)^+^, found 232.1298. [α]_D_^20^ = +8.0° (*c* 0.16, CH_2_Cl_2_).

##### *Tert*-butyl (1*R*)-1,2-dicyanoethylcarbamate (7)

Synthesized according to the *General procedure for the conversion of amides to nitriles*. White amorphous powder, yield: 76%; m.p. 133–136 °C. ^1^H-NMR (DMSO-d_6_, 400 MHz): δ = 1.43 (s, 9H, 3CH_3_), 3.11–3.14 (m, 2H, CH_2_-β-Asp), 4.91–4.96 (m, 1H, CH), 8.11 (d, 1H, *J* = 8.0 Hz, NH) ppm. MS (ESI): *m/z* (%) = 194.1 (M−H)^−^. IR (KBr): ν = 3358, 2984, 2941, 2258, 1685, 1517, 1372, 1340, 1311, 1280, 1158, 1057, 875, 604 cm^−1^. HRMS Calcd for C_9_H_12_N_3_O_2_ *m*/*z*: 194.0930 (M−H)^−^, found 194.0933. Anal. Calcd for C_9_H_13_N_3_O_2_ × 0.15 H_2_O (%): C, 54.62; H, 6.77; N, 21.23. Found: C, 54.61; H, 6.79; N, 21.22. [α]_D_^20^ = +31.1° (*c* 0.20, CH_2_Cl_2_).

##### *Tert*-butyl (3*R*)-2,5-bis(hydroxyimino)pyrrolidinylcarbamate (8)

Synthesized according to the *General procedure for the synthesis of cyclic imide dioximes*. Beige amorphous powder, yield: 93%; m.p. 223–226 °C. ^1^H-NMR (DMSO-d_6_, 400 MHz): δ = 1.38 (s, 9H, 3CH_3_), 2.40–2.45 (m, 1H, CH_2A_-β-Asp), 2.85–2.92 (m, 1H, CH_2B_-β-Asp), 4.64–4.70 (m, 1H, CH), 7.35 (d, 1H, *J* = 8.8 Hz, Boc-NH), 8.18 (s, 1H, NH), 9.68 (s, 1H, OH), 9.87 (s, 1H, OH) ppm. ^13^C-NMR (DMSO-d_6_, 100 MHz): δ = 28.2(C(CH_3_)_3_), 30.9 (CH_2_-β), 46.4 (CH), 78.1 (C(CH_3_)_3_), 148.0 (C=N), 150.4 (C=N), 154.9 (CO) ppm. MS (ESI): *m*/*z* (%) = 245.1 (M+H)^+^. IR (KBr): ν = 3449, 3349, 3203, 2883, 2362, 1683, 1524, 1370, 1318, 1273, 1253, 1167, 1061, 969, 939, 847, 790, 716 cm^−1^. HRMS Calcd for C_9_H_17_N_4_O_4_ *m*/*z*: 245.1219 (M+H)^+^, found 245.1213. Anal. Calcd for C_9_H_16_N_4_O_4_ × 0.1 H_2_O (%): C, 43.93; H, 6.64; N, 22.77. Found: C, 44.16; H, 6.41; N, 22.49. [α]_D_^20^ = +3.7° (*c* 0.14, CH_2_Cl_2_).

##### *Tert*-butyl (1*S*)-2-{[(1*R*)-1,3-dicyanopropyl]amino}-1-methyl-2-oxoethylcarbamate (9)

To an ice-chilled stirred mixture of trifluoroacetic acid and dichloromethane (*v*/*v* 1/5, 5 mL) Boc-protected compound (0.5 mmol) **2** was added and the mixture allowed to warm to room temperature. After 3 hours the reaction was completed and the solvent was evaporated in vacuo. The residue was washed three times with diethyl ether giving sufficiently pure free amine which was dissolved in dichloromethane (2 mL). In a parallel reaction, to a stirred solution of Boc-l-Ala (0.5 mmol) in dry dichloromethane (10 mL), diisopropylethylamine (DIPEA) (1.50 mmol) and TBTU (0.55 mmol) were added. After stirring for 45 minutes the solution of free amine in dichloromethane was added at 0 °C, and the mixture was allowed to warm to room temperature and stirring then continued for 48 h. Upon completion, the reaction mixture was diluted with dichloromethane (30 mL) and then washed with 1 M HCl (2 × 10 mL), water (10 mL), saturated NaHCO_3_ solution (2 × 10 mL), water (10 mL) and then dried over anhydrous Na_2_SO_4_. The solvent was concentrated in vacuo and the residue was purified by flash silica gel column chromatography (gradient elution; starting eluent: chloroform/methanol 20:1 *v*/*v*) to afford compound **9**. Yellow oil, yield: 57%; ^1^H-NMR (CDCl_3_, 400 MHz): δ = 1.39 (t, 3H, *J* = 7.2 Hz, CH_3_-Ala), 1.44 (s, 9H, 3CH_3_), 2.16–2.31 (m, 2H, CH_2_-β-Glu), 2.55 (t, 2H, *J* = 7.2 Hz, CH_2_-γ-Glu), 4.09–4.18 (m, 1H, CH-Ala), 4.94–5.05 (m, 2H, CH-Glu, NH-Ala), 7.45 (d, 1H, *J* = 8.4 Hz, NH-Glu) ppm. ^13^C-NMR (CDCl_3_, 100 MHz): δ = 13.7 (CH_2_-γ), 17.5 (CH_3_CH), 28.3 (C(CH_3_)_3_), 28.5 (CH_2_-β), 39.3 (CHCN), 50.2 (CHCH_3_), 80.9 (C(CH_3_)_3_), 117.2 (CN), 117.9 (CN), 156.0 (CO), 173.2 (CO) ppm. MS (ESI): *m/z* (%) = 279.1 (M−H)^−^. HRMS Calcd for C_13_H_19_N_4_O_3_ *m/z*: 279.1457 (M−H)^−^, found 279.1454. Anal. Calcd for C_13_H_20_N_4_O_3_ (%): C, 55.70; H, 7.19; N, 19.99. Found: C, 55.26; H, 6.80; N, 20.16. [α]_D_^20^ = −3.0° (*c* 0.14, CH_2_Cl_2_). 

##### *Tert*-butyl ((*S*)-1-(((*R*,*Z*)-6-(hydroxyamino)-2-(hydroxyimino)-2,3,4,5-tetrahydropyridin-3-yl)amino)-1-oxopropan-2-yl)carbamate (10)

Synthesized according to the *General procedure for the synthesis of cyclic imide dioximes*. Colorless oil, yield: 68%; ^1^H-NMR (DMSO-d_6_, 400 MHz): δ = 1.18 (t, 3H, *J* = 7.2 Hz, CH_3_-Ala), 1.37 (s, 9H, 3CH_3_), 1.68–1.76 (m, 1H, CH_2A_-β-Glu), 1.77–1.87 (m, 1H, CH_2B_-β-Glu), 3.96–3.98 (m, 1H, CH), 4.57–4.61 (m, 1H, CH), 6.83 (d, 1H, *J* = 7.6 Hz, Boc-NH), 8.26 (d, 1H, *J* = 8.0 Hz, CONH), 8.34 (s, 1H, NH), 10.00 (s, 1H, OH), 10.37 (s, 1H, OH) ppm. ^13^C-NMR (MeOD, 100 MHz): δ = 18.6 (CH_3_CH), 23.3 (CH_2_-β), 26.8 (CH_2_-γ), 28.7 (C(CH_3_)_3_), 46.8 (CHCH_3_), 51.8 (CH), 80.7 (C(CH_3_)_3_), 145.9 (C=N), 146.5 (C=N), 157.6 (CO), 175.5 (CO) ppm. MS (ESI): *m/z* (%) = 330.2 (M+H)^+^. IR (KBr): ν = 3322, 2980, 2603, 2496, 1666, 1616, 1526, 1397, 1365, 1320, 1242, 1162, 1072, 1032, 980, 926 cm^−1^. HRMS Calcd for C_13_H_23_N_5_O_5_ *m/z*: 330.1777 (M+H)^+^, found 330.1780. Anal. Calcd for C_13_H_23_N_5_O_5_ (%): C, 47.41; H, 7.04; N, 21.26. Found: C, 47.16; H, 6.81; N, 21.49. [α]_D_^20^ = −23.5° (*c* 0.22, MeOH). *CH_2_-γ-Glu hidden under the signal of DMSO.

### 2.2. Antioxidant Evaluation

#### 2.2.1. Ferrozine Assay

The ferrous chelating ability of novel imide dioximes was evaluated by measuring the formation of the ferrous ion-ferrozine complex [19]. Solutions of samples (15.7–500 µM) were prepared in a 1:1 (*v/v*) mixture of methanol and potassium acetate buffer (500 mM, pH 5.5). Freshly prepared water solution of FeCl_2_ (50 µL, 250 µM) or water (50 µL) was added to the 150 µL of either solution of the test sample (15.7–500 µM) or solvent (negative control) in a well. Quercetin and EDTA were used as positive control under the same assay conditions. Flat bottomed 96-well microtiter plate (TPP, Tissue Culture Test Plates) was shaken for 1 min and then left to stand at room temperature for 15 min. Solution of ferrozine (100 µL, 1 mM) in potassium acetate buffer (500 mM, pH 5.5) was added in each well. Microtiter plate was than incubated at room temperature in the dark. After 30 min, the absorbance was determined with a microplate reader (BioTek Synergy™ HT Multi-Detection Microplate Reader) at 562 nm. Experiments were performed in triplicate. The percentage of inhibition of iron(II)-ferrozine chelating ability was calculated by using the following equation: Percentage of I (%) = (A_0_ − (A_n_ − A_b_))/A_0_ × 100; where A_0_ is the absorbance of negative control, A_n_ is absorbance of the test sample and A_b_ absorbance of the sample without Fe^2+^. Inhibition of iron(II)-ferrozine chelating ability was expressed as the concentration of test sample that chelates 50% of Fe^2+^ ions (IC_50_) ± standard deviation.

#### 2.2.2. Ascorbate Redox System Assay

The ability of novel imide dioximes to halt copper and iron cycling was evaluated in ascorbate redox system assay as previously described [20]. All of the solutions, except CuSO_4_ and FeCl_3_ (dissolved and diluted in Milli-Q water only), were prepared in a phosphate (20 mM), NaCl (100 mM) buffer (PBS) at pH 7.4 with a final sample volume of 200 μL. Each experiment was performed in triplicate on flat bottomed 96-well microtiter plate (Greiner Bio-One). Hydroxyl radical production was measured as the conversion of coumarine-3-carboxylic acid (CCA) into 7-hydroxy-CCA (λ_excitation_ = 395 nm, λ_emission_ = 450 nm). The general order of addition was as follows: CCA (100 μM), ligand (compounds **3**, **I** or **II** at 30 μM or EDTA at 10 μM in the case of iron-ascorbate studies), copper (10 μM) or iron (10 μM), and then ascorbate (300 μM) after 30 min of incubation. All test solutions also contained 1 μM desferryl.

## 3. Results and Discussion

### 3.1. Synthesis

Amino acids were transformed to their corresponding cyclic imide dioximes as shown in Scheme 1. They were first *N*-protected by reaction with Boc-anhydride, then converted in very good yields (79–85% over two steps) to their corresponding diamides **1** [21] and **6**, using the [(Boc_2_)O]-(NH_4_)_2_CO_3_ system [22]. In the following step, cyanuric chloride in dimethylformamide at room temperature was used as a dehydrating agent [23,24] to convert the starting diamides quantitatively into their respective dinitriles **2** and **7**. This two-step sequence for the transformation of α-amino acids to α-aminonitriles, although rarely encountered in the literature, is very convenient and affords products with high enantiomeric purity. Subsequent treatment of the dinitriles **2** and **7** with hydroxylamine hydrochloride in the presence of triethylamine in refluxing ethanol [24,25], yielded the corresponding cyclic imide dioximes **3** and **8** in excellent yields (83–93%). A previously published report showed unambiguously that racemization, under the reaction conditions used for cyclization, does not take place [13]. Thus, we concluded that the stereochemistry of the starting amino acids was not compromised. Moreover, the obtained ^1^H and ^13^C NMR data were compared to the reported NMR data of the basic cyclic imide dioxime scaffolds **I** and **II**. The comparison has shown that the preferred tautomeric forms of **3** and **8** are those depicted in Scheme 1. The imide dioxime **3** was then treated with acetyl chloride affording the diacetylated product, compound **4**. Further, treatment of dinitrile **2** with hydroxylamine hydrochloride in an ethanol/water mixture, in the presence of sodium carbonate at room temperature [26], yielded bisamidoxime **5** in a good yield.

Next, we demonstrated that cyclic imide dioximes can be integrated into larger structures such as dipeptides (shown in Scheme 2). Thus, **2** underwent acidolytic removal of the *N*-Boc group, using a standard TFA-CH_2_Cl_2_ (1:5) protocol, yielding the free amine that immediately underwent TBTU-mediated coupling with Boc-L-Ala to afford compound **9**. It was subsequently treated with hydroxylamine hydrochloride in the presence of triethylamine in refluxing ethanol to afford Compound **10**, which consists of a glutarimide dioxime moiety linked to L-Ala. All the structures were fully characterized by spectroscopic data.

### 3.2. Evaluation of Iron-Chelating Properties

The compounds were first evaluated for their iron(II)-chelating abilities in the ferrozine assay and compared to that of quercetin, a known iron(II)-chelating agent (IC_50_ = 129 ± 15 µM) [14]. Ferrozine can quantitatively form complexes with iron(II); however, in the presence of chelating agents, the complex formation is disrupted, which can be measured spectrophotometrically [19,27,28]. The results obtained provided an insight into the structural requirements for iron(II) chelation of our set of compounds (see Table 1).

Compound **3** (IC_50_ = 268 ± 37.7 µM) proved as the most potent of the series, its iron(II)-chelating ability being only slightly lower than that of quercetin (IC_50_ = 129 ± 15.0 µM). Evidently, the 6-membered glutarimide dioxime is pivotal for iron(II)-chelating properties (compounds **I** (IC_50_ = 601 ± 12.6 µM), **3** (IC_50_ = 268 ± 37.7 µM), and **4** (IC_50_ = 375 ± 33.4 µM)), since the compounds carrying a 5-membered succinimide dioxime in their structure were devoid of any activity (compounds **II** and **8**). Further, the carbamate functionality appended to the 6-membered heterocycle enhances the Fe(II)-chelating properties (compound **3** (IC_50_ = 268 ± 37.7 µM)). Interestingly, the free oxime =N-OH groups do not seem to be important for complexing Fe(II), since the diacetylation of those groups led only to a minor drop in activity (compound **4** (IC_50_ = 375 ± 33.4 µM)). Moreover, the acyclic analogue of **3**, compound **5**, retains some of the Fe(II)-chelating ability (IC_50_ = 671 ± 30.7 µM), however the latter is significantly reduced when compared to that of the parent compound. The acylation of the free amine, however, resulted in compound **10**, which exhibits weak iron(II)-chelating activity (IC_50_ = 895 ± 33.1 µM).

### 3.3. Evaluation of Copper and Iron Cycling Inhibition in Ascorbate Redox System Assay

**Figure 3:**
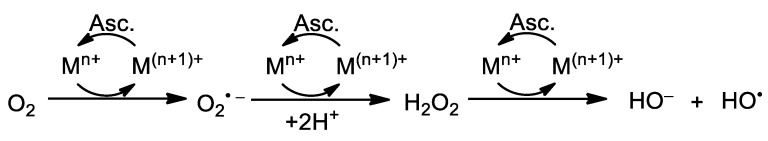
Production of hydroxyl radicals (HO^•^) by metal redox cycling in the presence of oxygen and ascorbate (Asc.). Metal ion (M^n+^) corresponds to Cu^+^ or Fe^2+^.

Selected compounds (**3**, **I** and **II**) were then evaluated for their anti-Fenton properties. To shed some light on the mechanism by which these compounds can inhibit Fenton activity, their ability to halt copper and iron cycling via metal complexation was assessed in the ascorbate redox system assay [29,30,31]. Fenton chemistry is characterized by the oxidation of redox-active metal ions in their reduced form (e.g., Fe(II) or Cu(I)) with hydrogen peroxide to generate hydroxyl radicals. The production of hydroxyl radicals (HO^•^) by metal redox cycling in the presence of oxygen and ascorbate (shown in Figure 3) was quantified by measuring the increase in fluorescence. The latter is a result of CCA, which generates fluorescent 7-hydroxy-CCA in the presence of hydroxyl radicals.

**Figure 4:**
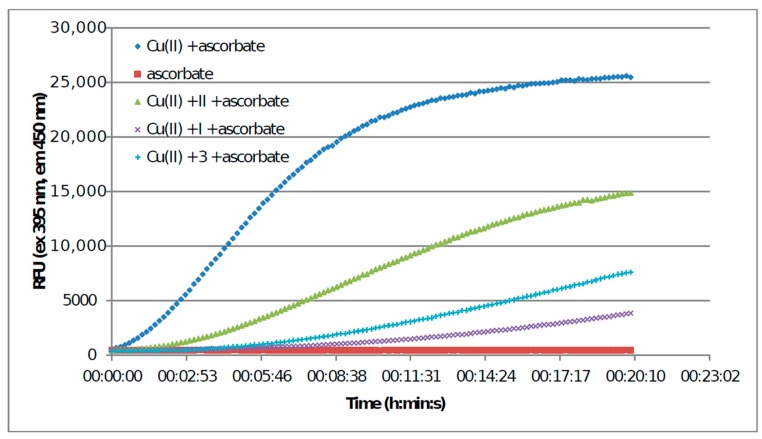
Fluorescence intensity of 7-hydroxy-CCA after incubation of CCA [100 µM] and Cu(II) [10 µM] with ascorbate [300 µM] (♦). Compound **II** (▲) [30 µM], **I** (×) [30 µM] or **3** (+) [30 µM] were added 30 min prior to ascorbate. Ascorbate (■) without Cu(II) was a negative control. All solutions except CuSO_4_ (dissolved in Milli-Q water only) were prepared in KH_2_PO_4_ [20 µM], NaCl [100 µM] buffer containing desferryl [1 µM], pH = 7.4.

**Figure 5:**
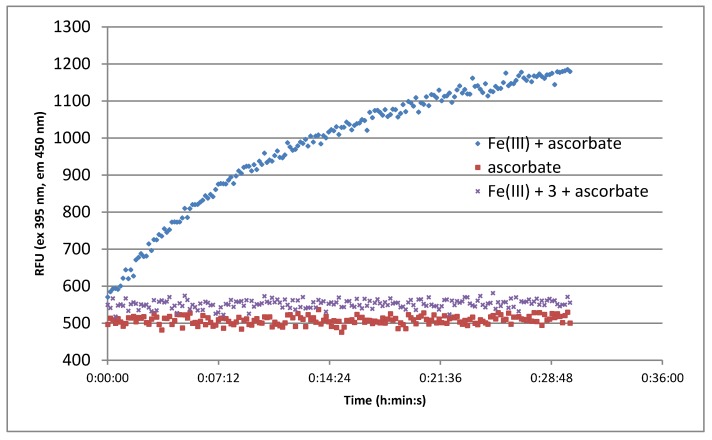
Fluorescence intensity of 7-hydroxy-CCA after incubation of CCA [100 µM] and Fe(III) [10 µM] with ascorbate [300 µM] (♦). Compound **3** (×) [30 µM] was added 30 min prior to ascorbate. Ascorbate (■) is a negative control with CCA, buffer, **3** and ascorbate without Fe(III). All solutions except FeCl_3_ (dissolved in Milli-Q water only) were prepared in KH_2_PO_4_ [20 µM], NaCl [100 µM] buffer containing desferryl [1 µM], pH = 7.4.

As shown in Figure 4, the fluorescence of 7-hydroxy-CCA in the copper-ascorbate system increases steadily with time and reaches a plateau after approximately 10 minutes. When compounds **3**, **I** or **II** were co-incubated with the copper-ascorbate system, this process was substantially slowed down (also shown in Supporting information File 1; Figures S1–S3). As the redox-active copper ions have been related to the production of ROS, this indicates that all tested ligands were able to halt the copper redox cycling by metal complexation. Compounds **3** and **I**, carrying a glutarimide dioxime motif in their structures, exhibited the strongest inhibitory capacity, while compound **II** incorporating a succinimide dioxime moiety in its structure exhibited only a moderate inhibition of copper redox cycling. Incidentally, **I** has previously been demonstrated to form a strong complex with Cu(II) [3]. The fluorescence of 7-hydroxy-CCA in the iron-ascorbate system increases much slower with time (Figure 5) compared to copper-ascorbate system. Fe-EDTA system was employed as positive control to promote the Fenton reaction [14]. In agreement with previous findings, EDTA complexation of iron considerably increased the production rate of hydroxyl radicals (shown in Supporting information File 1; Figures S4, S6 and S8). On the other hand, co-incubation of reaction mixture with compounds **I** or **II** had practically no effect on the hydroxyl production rate (shown in Supporting information File 1; Figures S7 and S9). This was surprising, given the fact that **I** has previously been shown to strongly bind Fe(III) [3]. However, when compound **3** was co-incubated with the iron-ascorbate system, the production of hydroxyl radicals was halted entirely (Figure 5 and Figure S5). These findings suggest an important role of iron(II) chelation in the anti-Fenton activity of compound **3**.

## 4. Conclusions

In conclusion, we have designed a novel route for preparing chiral cyclic imide dioximes starting from the readily available amino acids. The synthesized compounds constitute building blocks capable of being integrated into larger structures. Their iron(II)-chelating abilities have also been determined. The glutarimide dioxime moiety has been established as an iron(II)-binding motif and imparts potent antioxidant properties to the compounds. Compound **3** was shown to strongly suppress hydroxyl radical formation by preventing iron cycling via Fe-complexation. These findings provide valuable insights into the structural requirements for achieving anti-Fenton activity and highlight the potential use of glutarimide dioximes as antioxidants.

## Figures and Tables

**Table 1:** Iron(II)-chelating properties of the synthesized compounds.

Compound	IC_50_ (Ferrozine Assay) ± S.D. [µM]
**3**	268 ± 37.7
**4**	375 ± 33.4
**5**	671 ± 30.7
**8**	/
**10**	895 ± 33.1
**I**	601 ± 12.6
**II**	/
**Quercetin**	129 ± 15.0
**EDTA**	21.1 ± 0.7

## Supplementary Materials

Supplementary data related to this article are available online at https://www.mdpi.com/2076-3921/8/10/473/s1; it contains ^13^C NMR data for all new compounds and measurements of ferrous chelating ability (IC_50_) and ascorbate studies. Figure S1: Effect of compound **3** on copper redox cycling, Figure S2: Effect of compound **I** on copper redox cycling, Figure S3: Effect of compound **II** on copper redox cycling, Figure S4: Effects of compound **3** and EDTA on iron redox cycling, Figure S5: Effect of compound **3** on iron redox cycling, Figure S6: Effects of compound **I** and EDTA on iron redox cycling, Figure S7: Effect of compound **I** on iron redox cycling, Figure S8: Effects of compound **II** and EDTA on iron redox cycling, Figure S9: Effect of compound **II** on iron redox cycling.
