# Supplementary material for: Iron-Binding and Anti-Fenton Properties of Novel Amino Acid-Derived Cyclic Imide Dioximes
Source: Antioxidants (Basel). 2019 Oct 11;8(10):473. doi: 10.3390/antiox8100473 (PMC6826749; doi:10.3390/antiox8100473)
Supplement: Supplementary file 1 [file antioxidants-08-00473-s001.pdf]

SUPPORTING INFORMATION

Iron-binding and anti-Fenton properties of novel amino acid-derived cyclic imide dioximes

*Janez Mravljak and Žiga Jakopin**

Faculty of Pharmacy, University of Ljubljana, Aškerčeva 7, SI – 1000 Ljubljana, Slovenia.

*Corresponding author:

Žiga Jakopin

Phone: +386 1 4769 646

Fax: + 386 1 4258 031

E-mail address: ziga.jakopin@ffa.uni-lj.si

Table of Contents:

1. ^{1}H, ^{13}C NMR and HRMS Spectra of compound **3**
2. ^{13}C NMR Spectra of selected compounds
3. The ferrous chelating ability – titration curves
4. Ascorbate studies

1. ^{1}H, ^{13}C NMR and HRMS Spectra of compound 3

Tert-butyl (3R)-2,6-bis(hydroxyimino)piperidinylcarbamate (**3**)

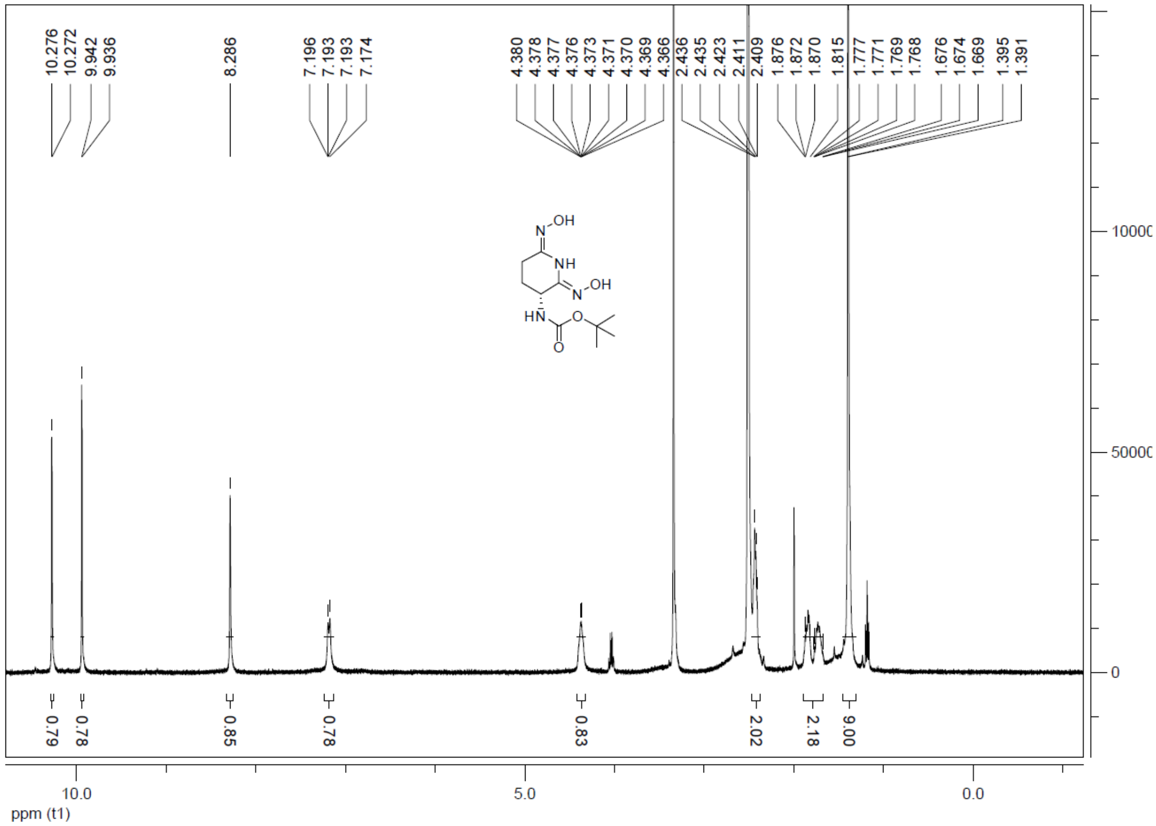

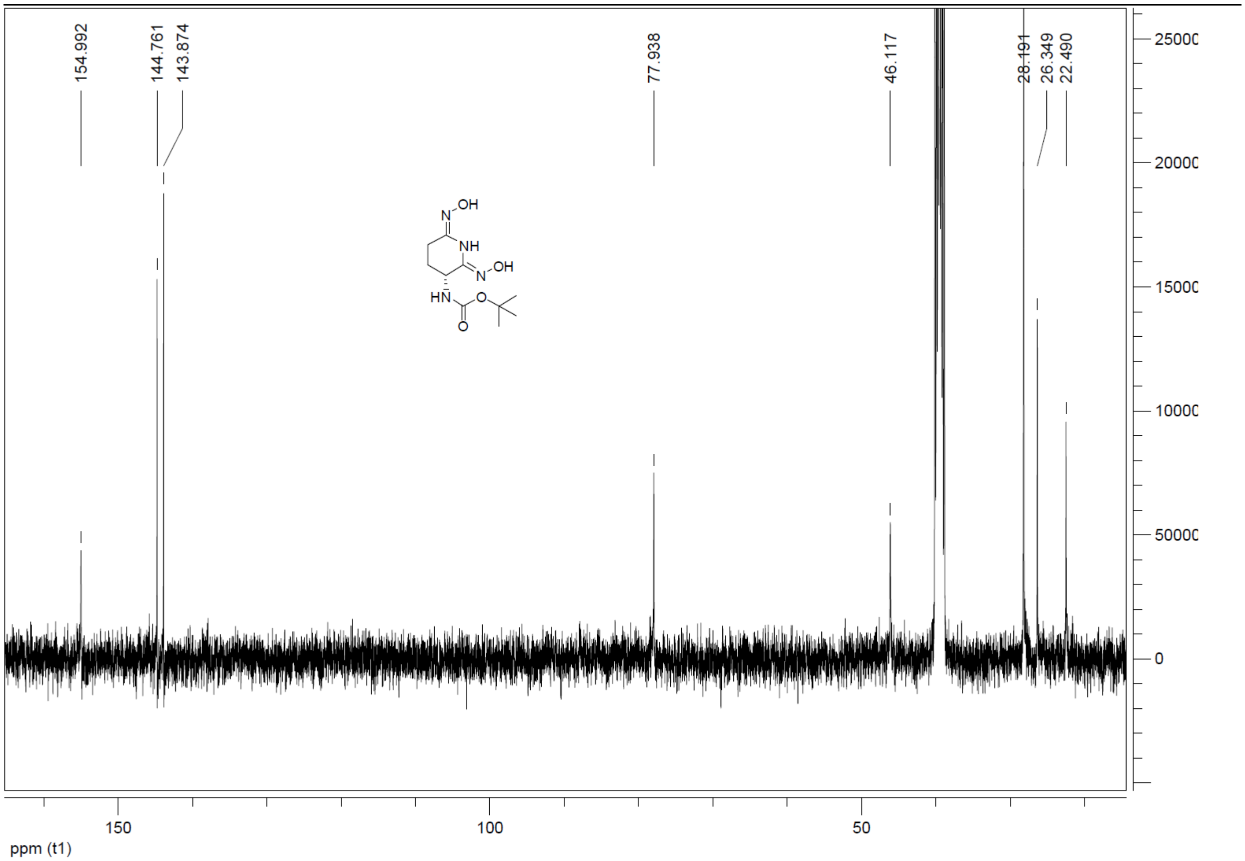

Elemental Composition Report

Single Mass Analysis

Tolerance = 20.0 PPM / DBE: min = -1.5, max = 100.0
Element prediction: Off
Number of isotope peaks used for i-FIT = 3

Monoisotopic Mass, Even Electron Ions
417 formula(e) evaluated with 7 results within limits (up to 20 best isotopic matches for each mass)
Elements Used:
C: 0-500 H: 0-1000 N: 0-200 O: 0-200
ZJ 70 7 (0.332) Cm (5:10)
1: TOF MS ES+

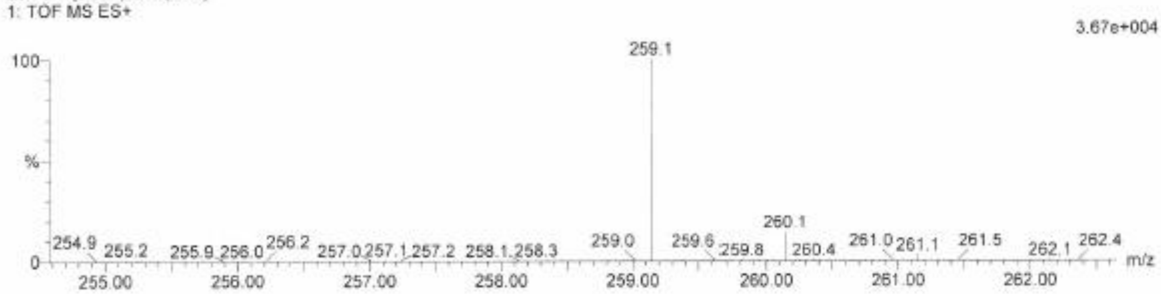

Mass	Calc. Mass	mDa	PPM	DBE	i-FIT	i-FIT (Norm)	Formula
Minimum:				-1.5			
Maximum:		5.0	20.0	100.0			
259.1398	259.1406	-0.8	-3.1	3.5	376.8	0.7	C10 H19 N4 O4
	259.1393	0.5	1.9	-1.5	377.5	1.5	C9 H23 O8
	259.1447	-4.9	-18.9	7.5	377.6	1.5	C15 H19 N2 O2
	259.1420	-2.2	-8.5	8.5	378.8	2.8	C11 H15 N8
	259.1379	1.9	7.3	4.5	381.2	5.2	C6 H15 N10 O2
	259.1366	3.2	12.3	-0.5	381.3	5.3	C5 H19 N6 O6
	259.1353	4.5	17.4	5.5	385.4	9.4	C2 H11 N16

2. ^{13}C NMR spectra of selected compounds

Tert-butyl ((R,2Z,6Z)-2,6-bis(acetoxyimino)piperidin-3-yl)carbamate (**4**)

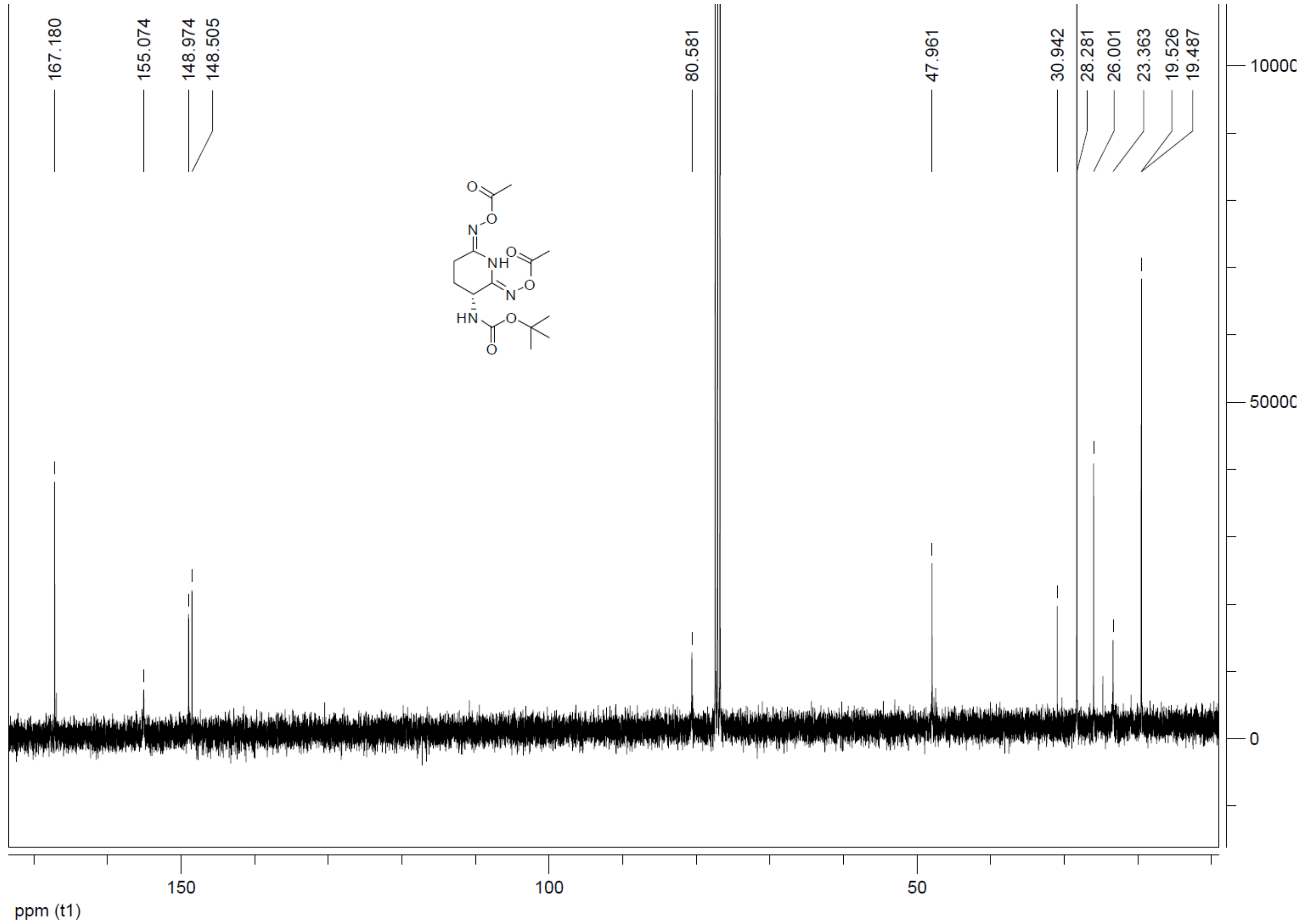

Tert-butyl ((R,1Z,5Z)-1,5-diamino-1,5-bis(hydroxyimino)pentan-2-yl)carbamate (**5**)

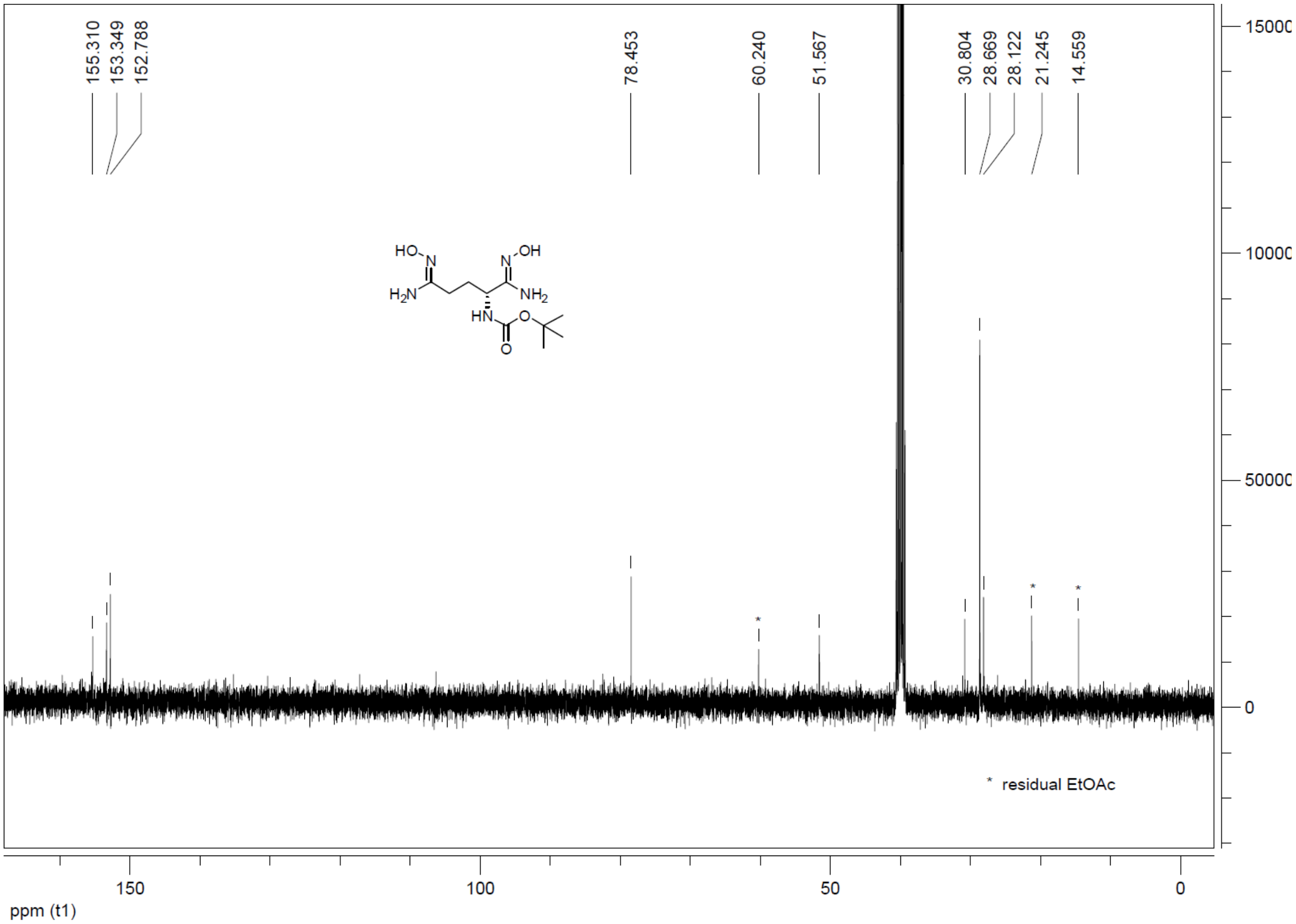

Tert-butyl (3R)-2,5-bis(hydroxyimino)pyrrolidinylcarbamate (**8**)

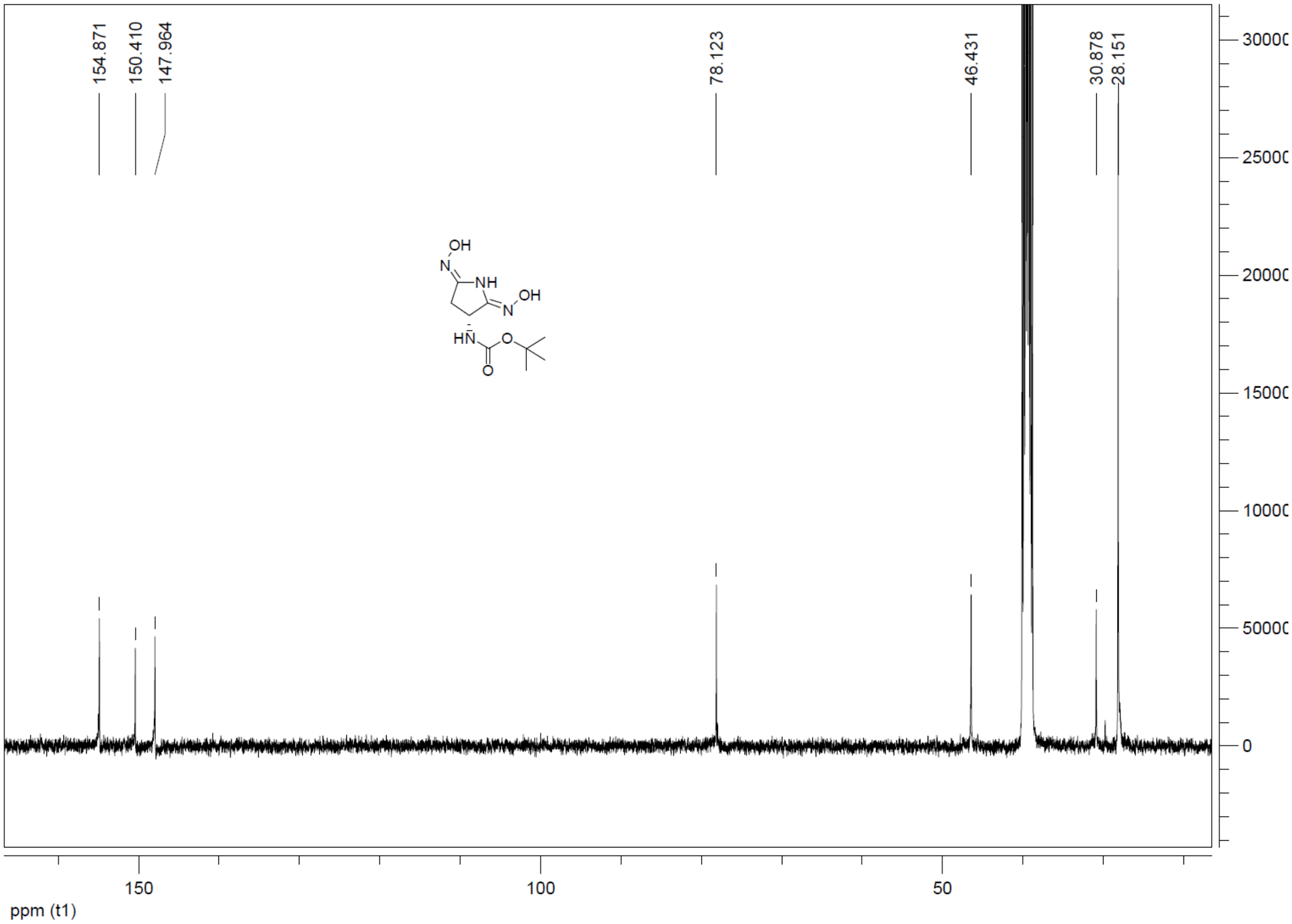

Tert-butyl (1S)-2-{[(1R)-1,3-dicyanopropyl]amino}-1-methyl-2-oxoethylcarbamate (**9**)

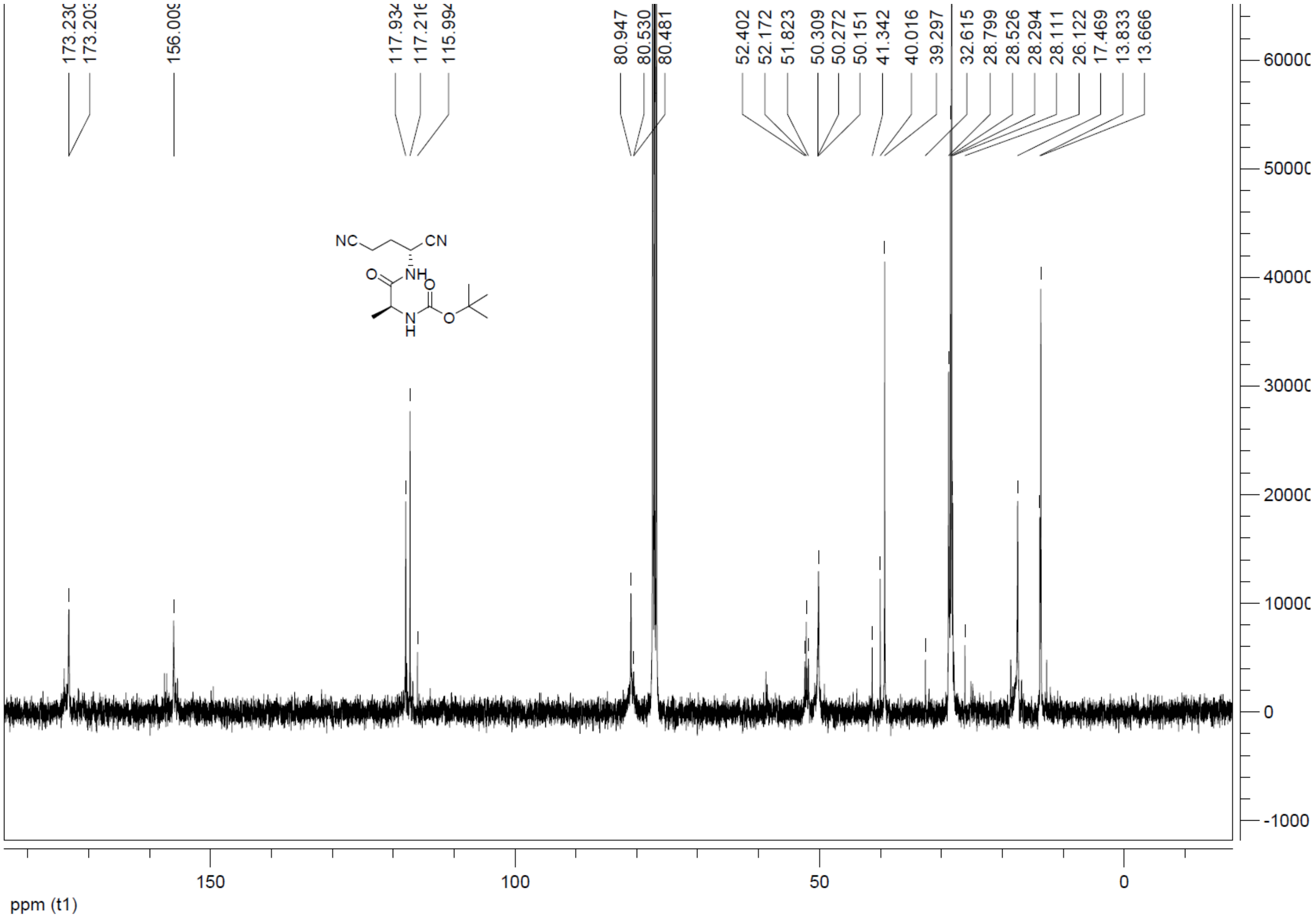

Tert-butyl ((S)-1-(((R,Z)-6-(hydroxyamino)-2-(hydroxyimino)-2,3,4,5-tetrahydropyridin-3-yl)amino)-1-oxopropan-2-yl)carbamate (**10**)

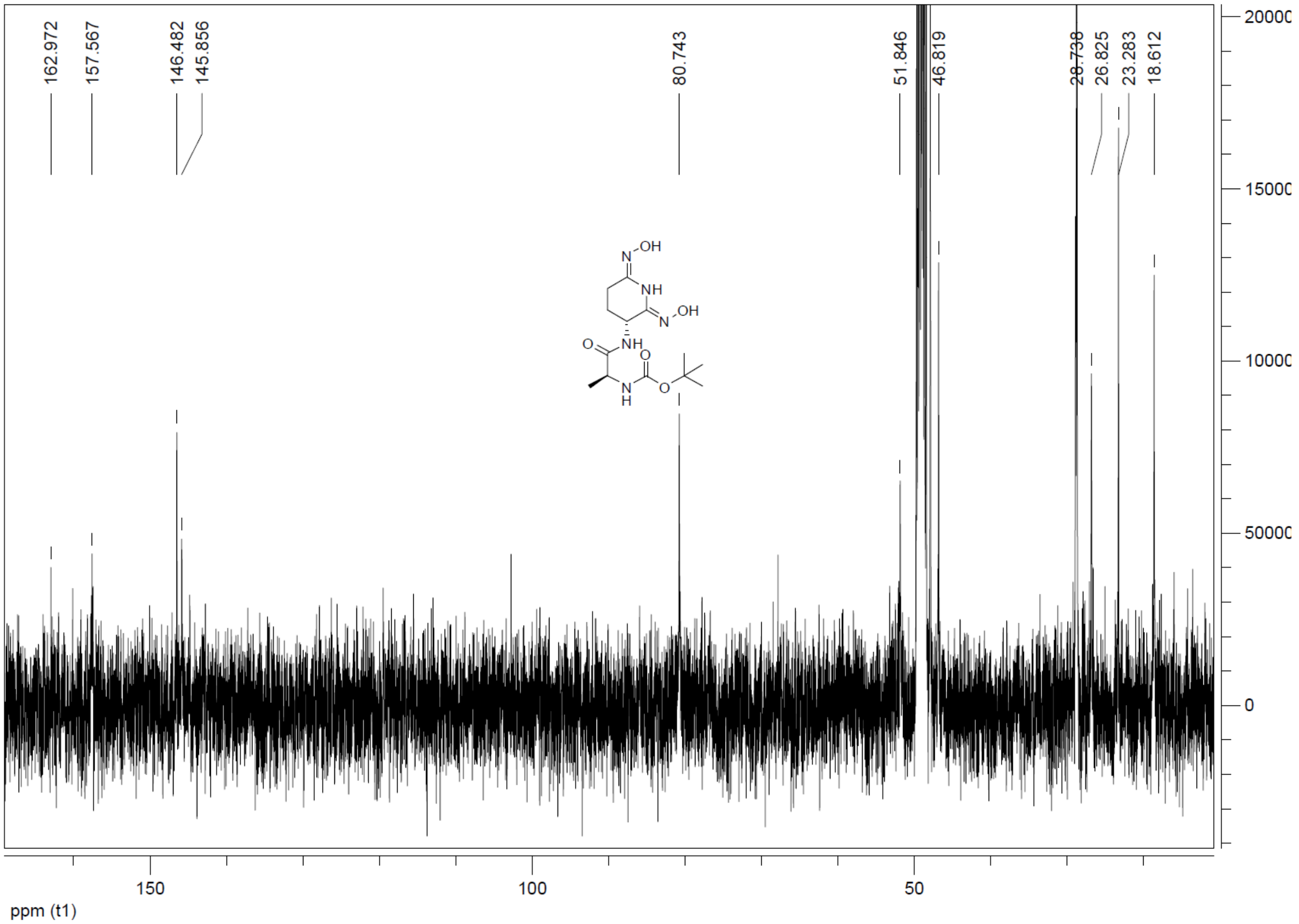

3. The ferrous chelating ability. The percentage of inhibition of iron(II)-ferrozine was plotted against concentration of sample **3**. Data are expressed as mean ± S.D. (n = 3).

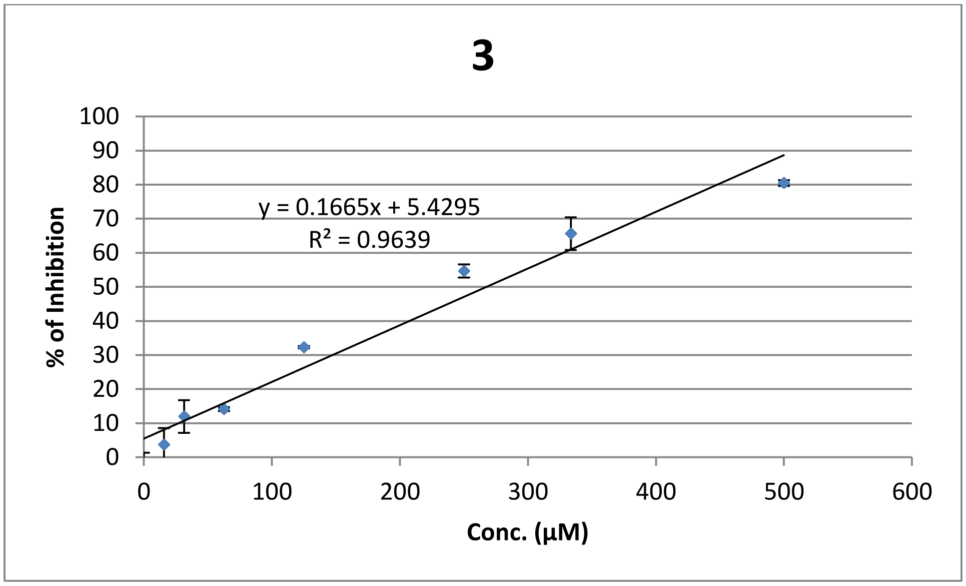

The ferrous chelating ability. The percentage of inhibition of iron(II)-ferrozine was plotted against concentration of sample **4**. Data are expressed as mean ± S.D. (n = 3).

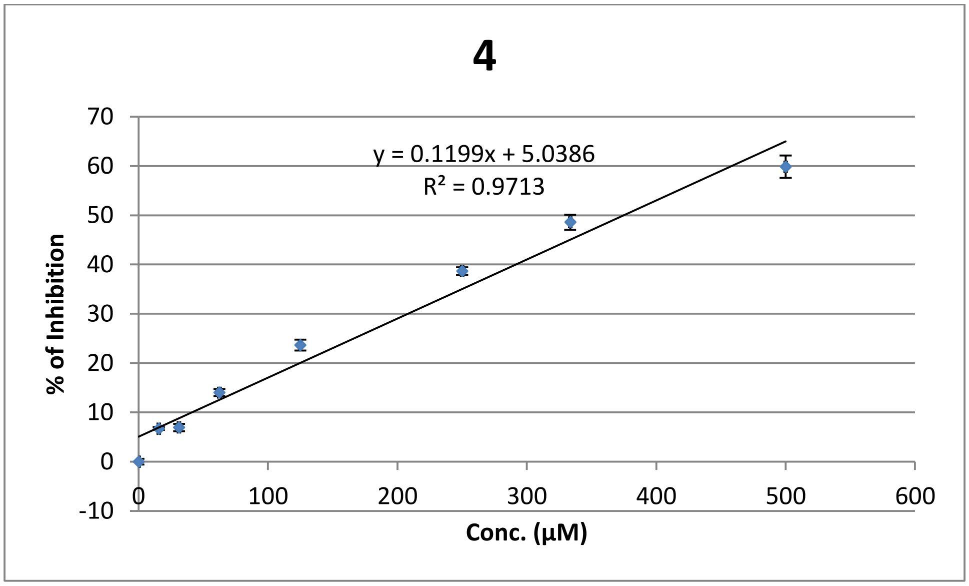

The ferrous chelating ability. The percentage of inhibition of iron(II)-ferrozine was plotted against concentration of sample **5**. Data are expressed as mean ± S.D. (n = 3).

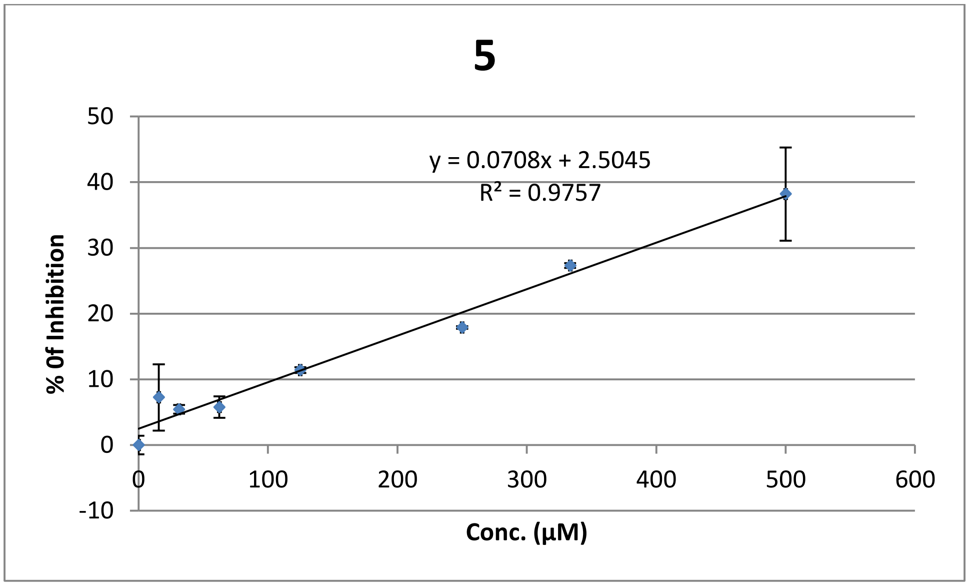

The ferrous chelating ability. The percentage of inhibition of iron(II)-ferrozine was plotted against concentration of sample **8**. Data are expressed as mean ± S.D. (n = 3).

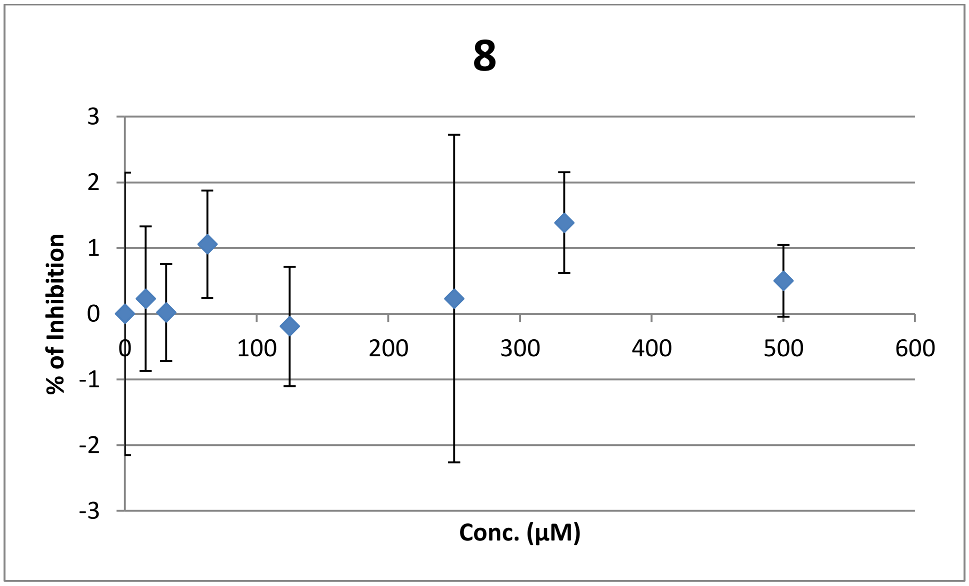

The ferrous chelating ability. The percentage of inhibition of iron(II)-ferrozine was plotted against concentration of sample **10**. Data are expressed as mean ± S.D. (n = 3).

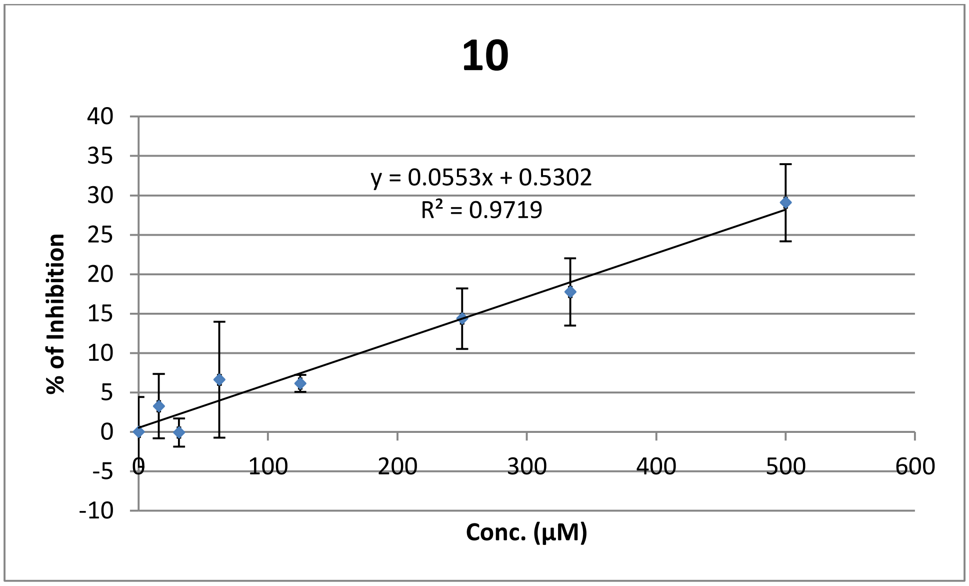

The ferrous chelating ability. The percentage of inhibition of iron(II)-ferrozine was plotted against concentration of sample **I**. Data are expressed as mean ± S.D. (n = 3).

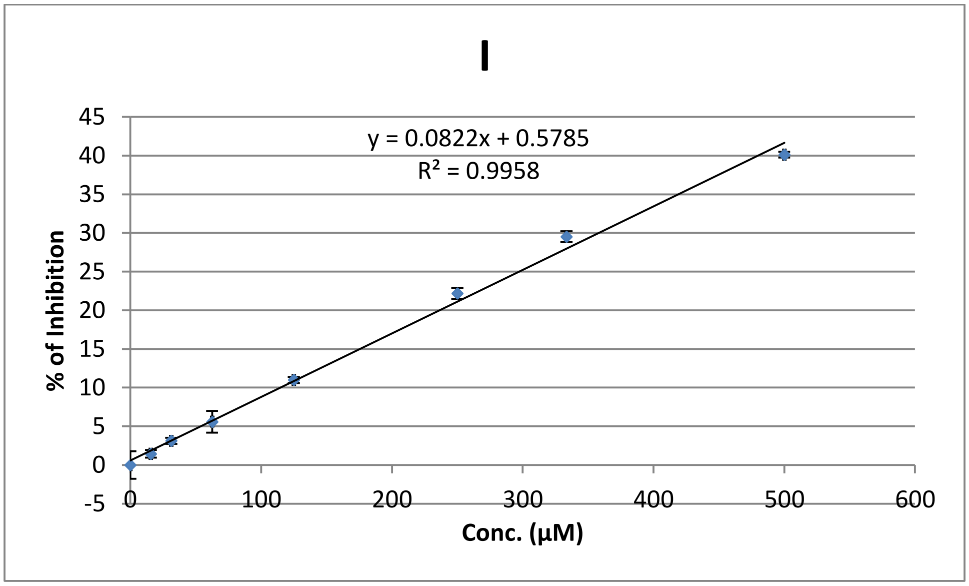

The ferrous chelating ability. The percentage of inhibition of iron(II)-ferrozine was plotted against concentration of sample **II**. Data are expressed as mean ± S.D. (n = 3).

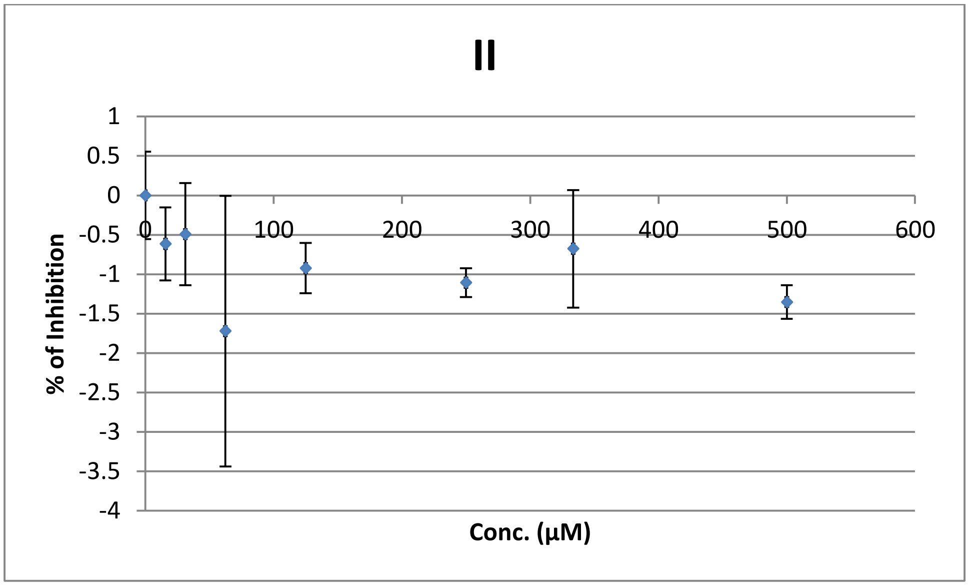

The ferrous chelating ability. The percentage of inhibition of iron(II)-ferrozine was plotted against concentration of **quercetin**. Data are expressed as mean ± S.D. (n = 3).

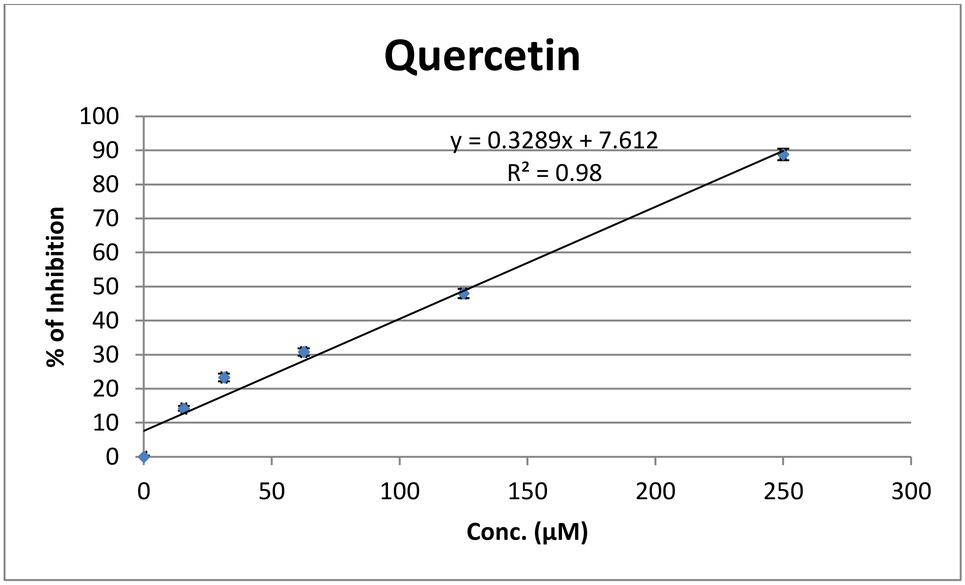

The ferrous chelating ability. The percentage of inhibition of iron(II)-ferrozine was plotted against concentration of **EDTA**. Data are expressed as mean ± S.D. (n = 3).

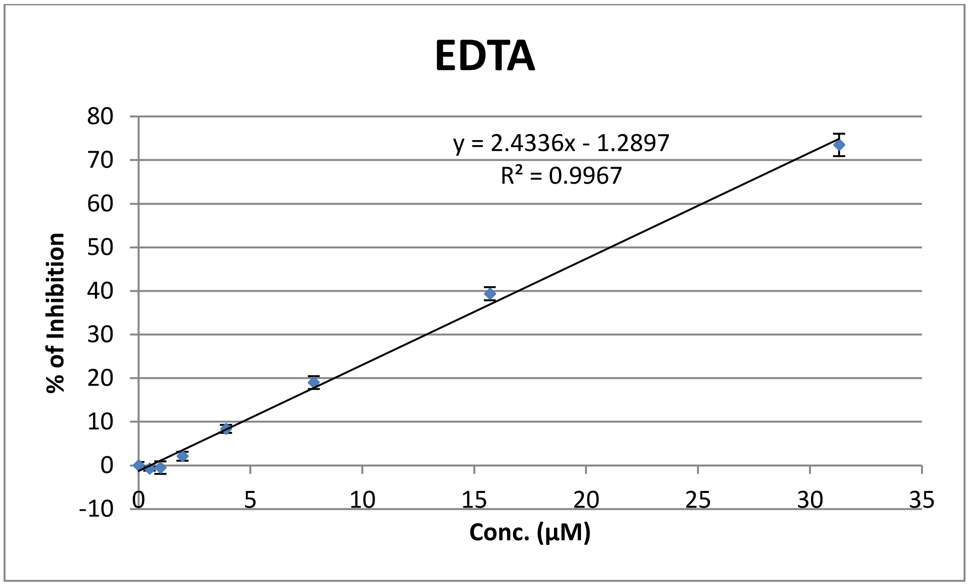

4. Ascorbate studies.

Figure S1. Effect of compound **3** on copper redox cycling. Fluorescence intensity of 7-hydroxy-CCA after incubation of CCA [100 μM] and Cu(II) [10 μM] with ascorbate [300 μM] (♦). Compound **3** (▲) [30 μM] was added 30 min prior to ascorbate. Ascorbate (■) is a negative control with CCA, buffer, **3** and ascorbate without Cu(II). All solutions except $CuSO_4$ (dissolved in Milli-Q water only) were prepared in KH_2PO_4 [20 μM], NaCl [100 μM] buffer containing desferryl [1 μM], pH = 7.4.

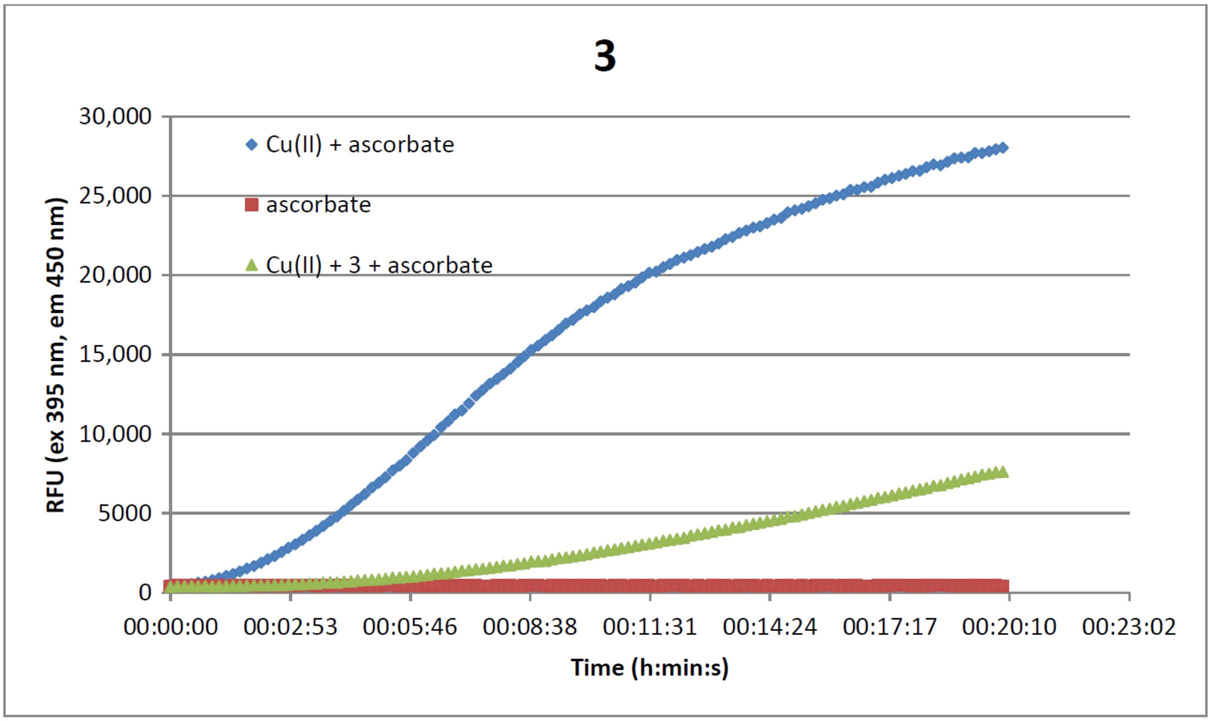

Figure S2. Effect of compound **I** on copper redox cycling. Fluorescence intensity of 7-hydroxy-CCA after incubation of CCA [100 μM] and Cu(II) [10 μM] with ascorbate [300 μM] (♦). Compound **I** (▲) [30 μM] was added 30 min prior to ascorbate. Ascorbate (■) is a negative control with CCA, buffer, **I** and ascorbate without Cu(II). All solutions except $CuSO_4$ (dissolved in Milli-Q water only) were prepared in KH_2PO_4 [20 μM], NaCl [100 μM] buffer containing desferryl [1 μM], pH = 7.4.

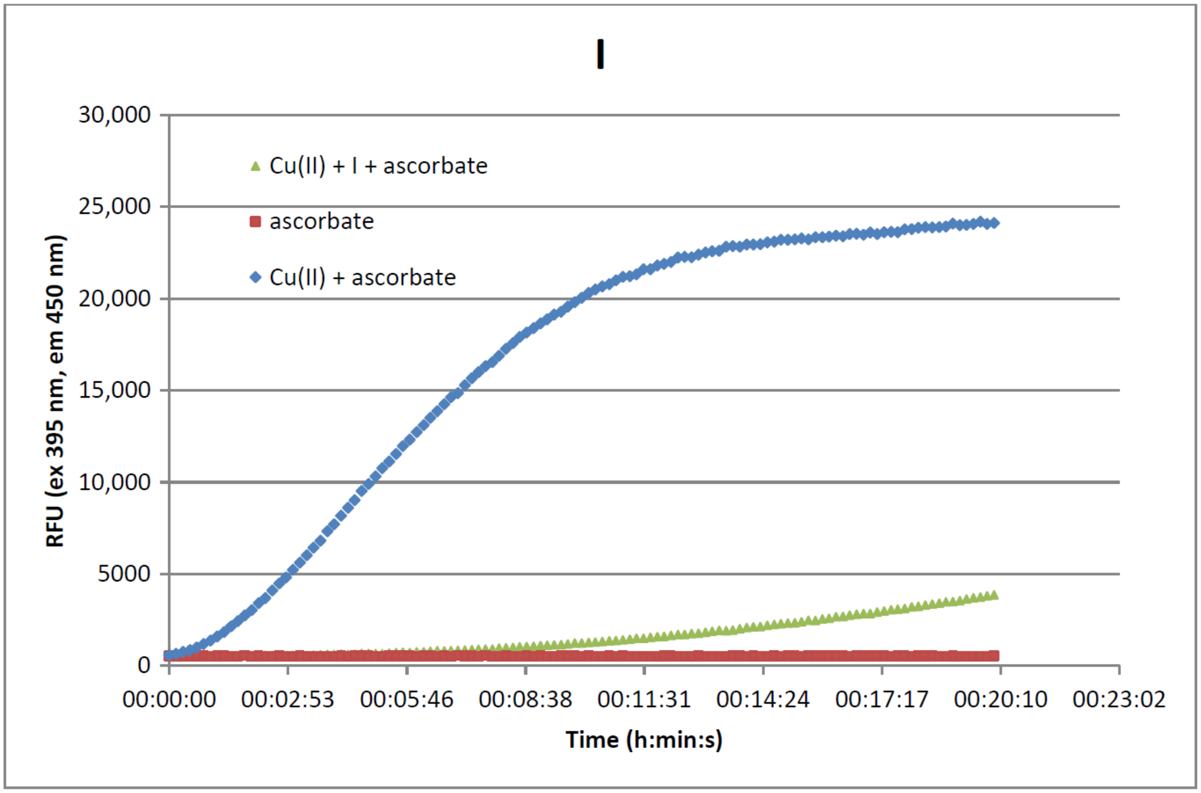

Figure S3. Effect of compound **II** on copper redox cycling. Fluorescence intensity of 7-hydroxy-CCA after incubation of CCA [100 μM] and Cu(II) [10 μM] with ascorbate [300 μM] (♦). Compound **II** (▲) [30 μM] was added 30 min prior to ascorbate. Ascorbate (■) is a negative control with CCA, buffer, **II** and ascorbate without Cu(II). All solutions except $CuSO_4$ (dissolved in Milli-Q water only) were prepared in KH_2PO_4 [20 μM], NaCl [100 μM] buffer containing desferryl [1 μM], pH = 7.4.

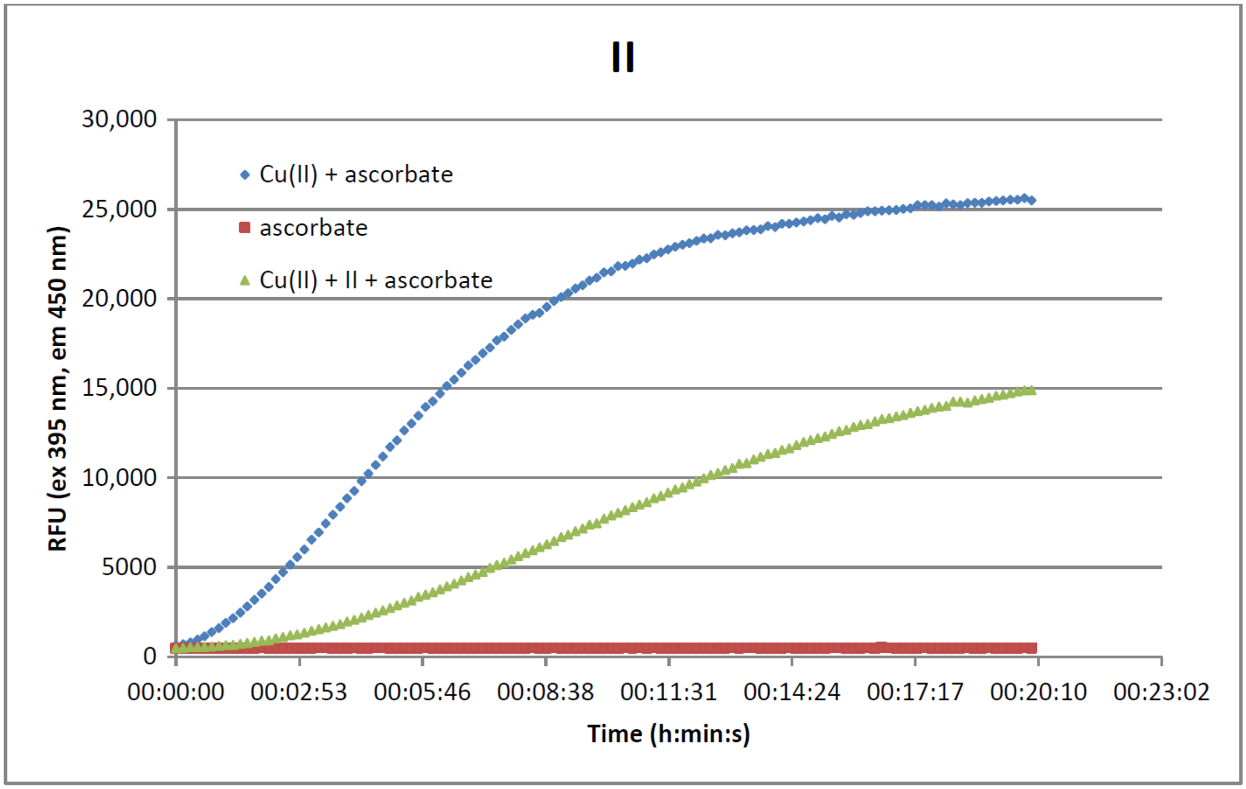

Figure S4. Effects of compound **3** and EDTA on iron redox cycling. Fluorescence intensity of 7-hydroxy-CCA after incubation of CCA [100 μM] and Fe(III) [10 μM] with ascorbate [300 μM] (♦). EDTA (▲) [10 μM] or compound **3** (×) [30 μM] was added 30 min prior to ascorbate. Ascorbate (■) is a negative control with CCA, buffer, **3** and ascorbate without Fe(III). All solutions except $FeCl_3$ (dissolved in Milli-Q water only) were prepared in KH_2PO_4 [20 μM], NaCl [100 μM] buffer containing desferryl [1 μM], pH = 7.4.

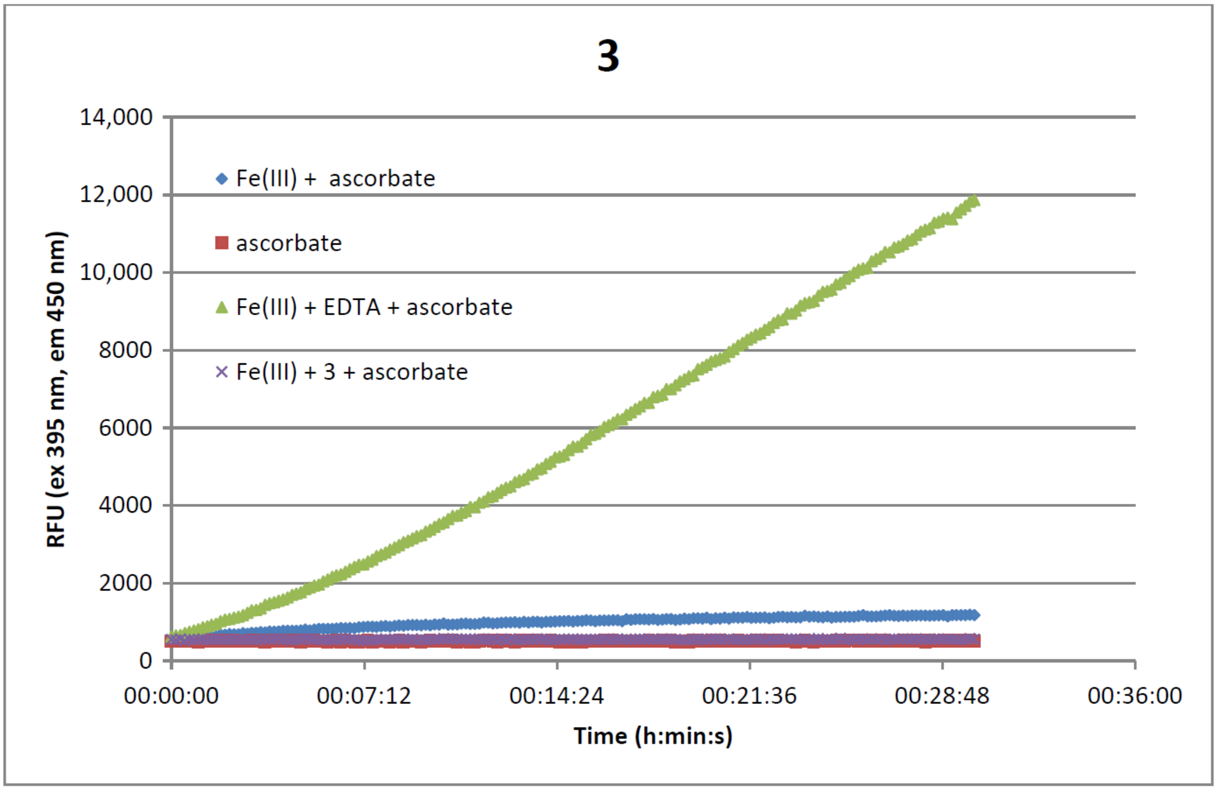

Figure S5. Effect of compound **3** on iron redox cycling. Same as above without the depiction of the curve for EDTA:

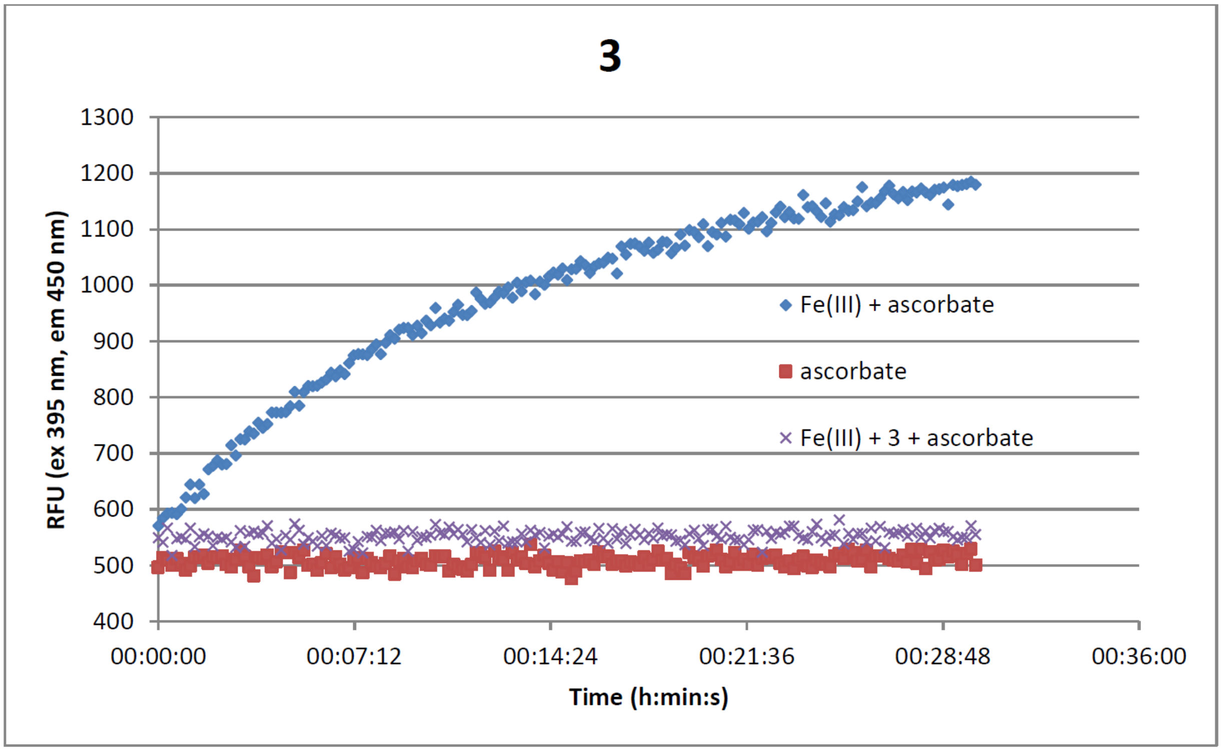

3
1300
1200
1100
1000
900
800
700
600
500
400
RFU (ex 395 nm, em 450 nm)
00:00:00
00:07:12
00:14:24
00:21:36
00:28:48
00:36:00
Time (h:min:s)
Fe(III) + ascorbate
ascorbate
Fe(III) + 3 + ascorbate

Figure S6. Effects of compound **I** and EDTA on iron redox cycling. Fluorescence intensity of 7-hydroxy-CCA after incubation of CCA [100 μM] and Fe(III) [10 μM] with ascorbate [300 μM] (♦). EDTA (▲) [10 μM] or compound **I** (×) [30 μM] was added 30 min prior to ascorbate. Ascorbate (■) is a negative control with CCA, buffer, **I** and ascorbate without Fe(III). All solutions except $FeCl_3$ (dissolved in Milli-Q water only) were prepared in KH_2PO_4 [20 μM], NaCl [100 μM] buffer containing desferryl [1 μM], pH = 7.4.

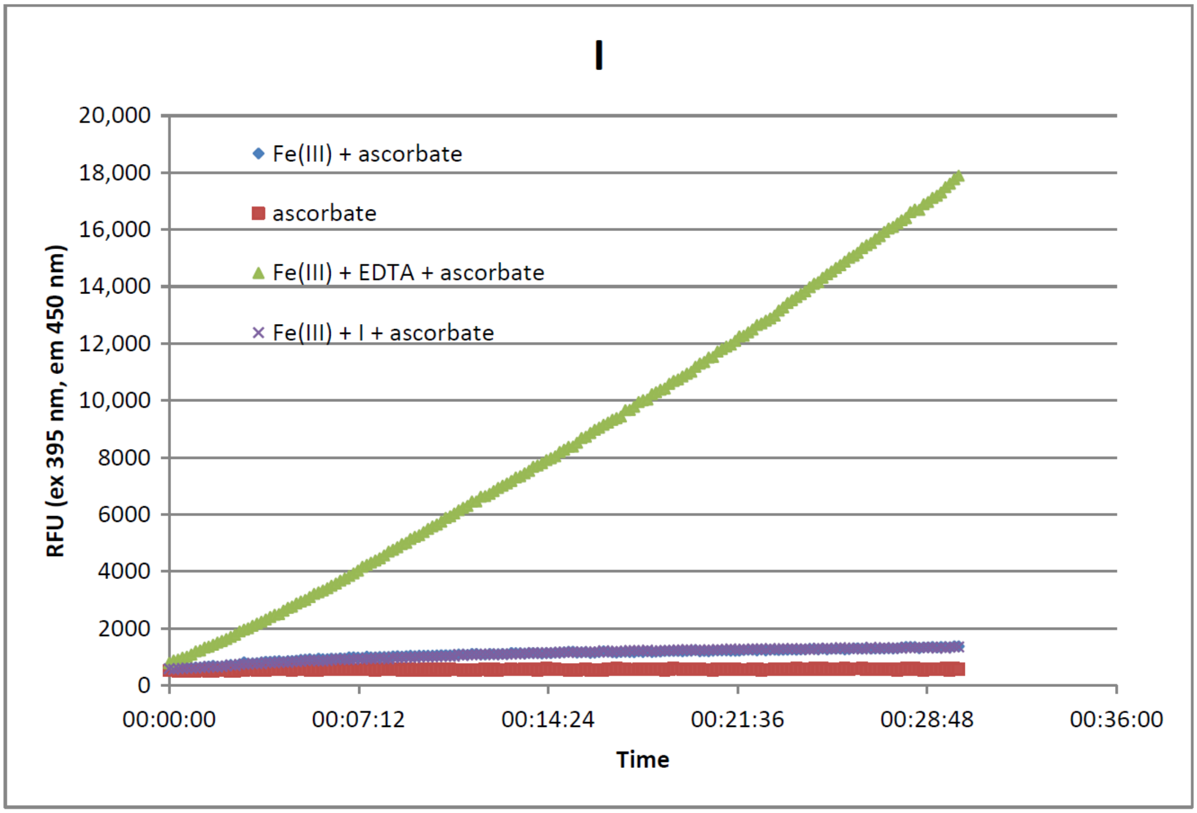

Figure S7. Effect of compound **I** on iron redox cycling. Same as above without the depiction of the curve for EDTA:

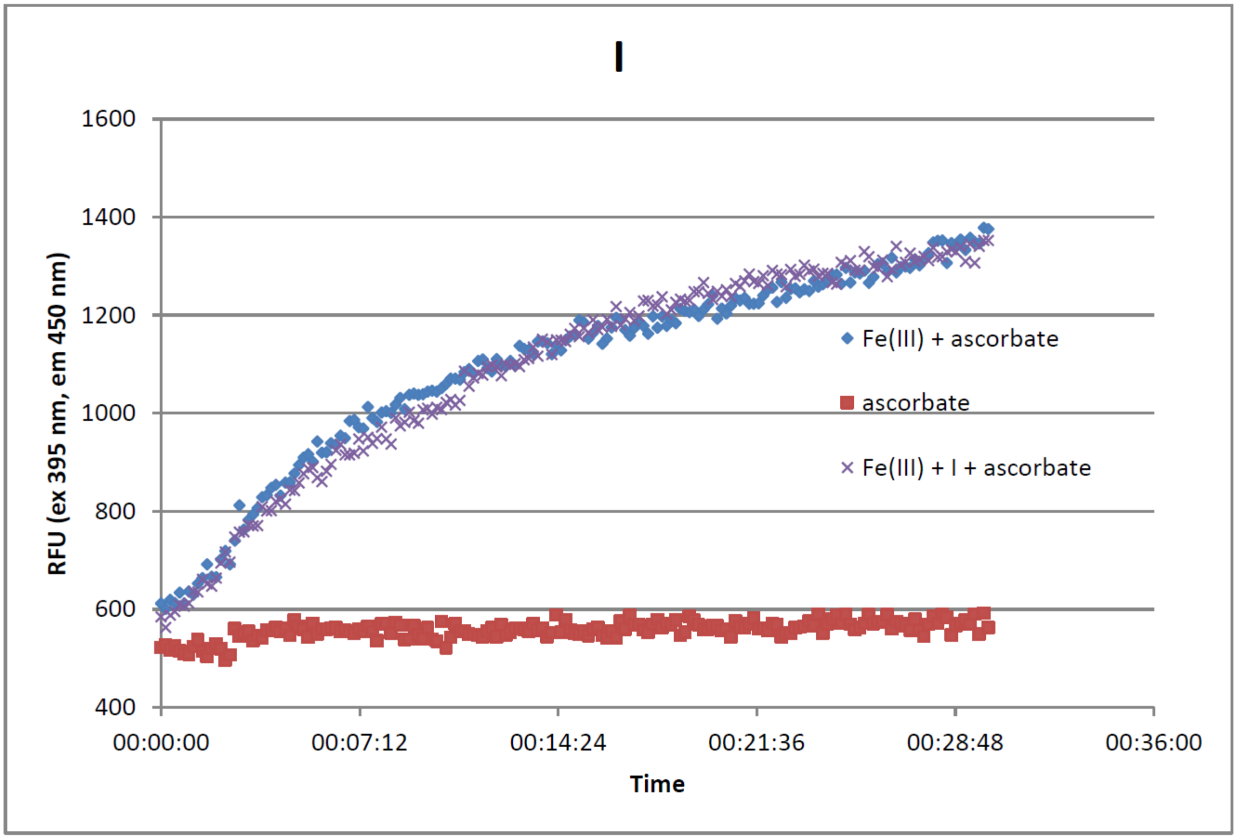
I
1600
1400
1200
1000
800
600
400
RFU (ex 395 nm, em 450 nm)
00:00:00
00:07:12
00:14:24
00:21:36
00:28:48
00:36:00
Time
Fe(III) + ascorbate
ascorbate
Fe(III) + I + ascorbate

Figure S8. Effects of compound **II** and EDTA on iron redox cycling. Fluorescence intensity of 7-hydroxy-CCA after incubation of CCA [100 µM] and Fe(III) [10 µM] with ascorbate [300 µM] (♦). EDTA (▲) [10 µM] or compound **II** (×) [30 µM] was added 30 min prior to ascorbate. Ascorbate (■) is a negative control with CCA, buffer, **II** and ascorbate without Fe(III). All solutions except $FeCl_3$ (dissolved in Milli-Q water only) were prepared in KH_2PO_4 [20 µM], NaCl [100 µM] buffer containing desferryl [1 µM], pH = 7.4.

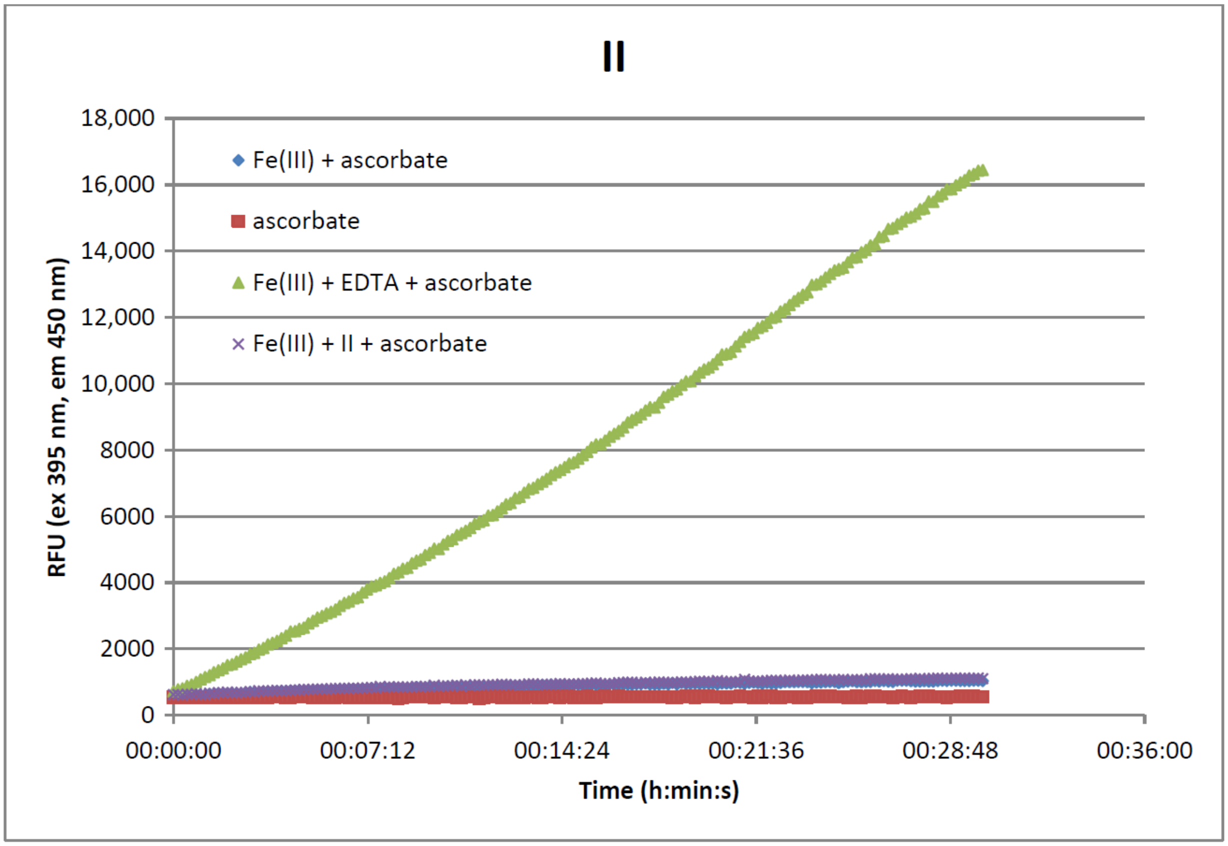

Figure S9. Effect of compound **II** on iron redox cycling. Same as above without the depiction of the curve for EDTA:

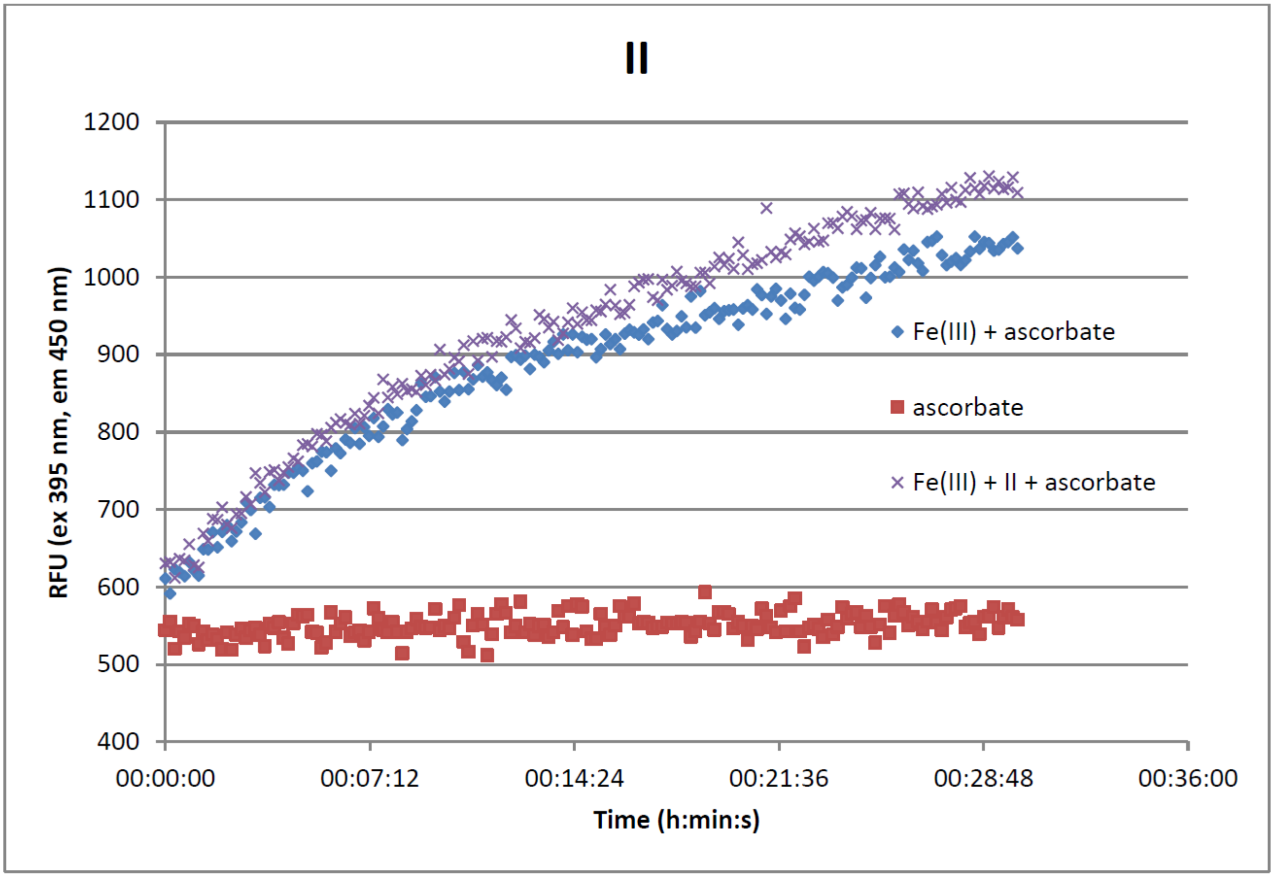
II
1200
1100
1000
900
800
700
600
500
400
RFU (ex 395 nm, em 450 nm)
00:00:00
00:07:12
00:14:24
00:21:36
00:28:48
00:36:00
Time (h:min:s)
Fe(III) + ascorbate
ascorbate
Fe(III) + II + ascorbate
